# High Levels of Diversity Uncovered in a Widespread Nominal Taxon: Continental Phylogeography of the Neotropical Tree Frog *Dendropsophus minutus*

**DOI:** 10.1371/journal.pone.0103958

**Published:** 2014-09-10

**Authors:** Marcelo Gehara, Andrew J. Crawford, Victor G. D. Orrico, Ariel Rodríguez, Stefan Lötters, Antoine Fouquet, Lucas S. Barrientos, Francisco Brusquetti, Ignacio De la Riva, Raffael Ernst, Giuseppe Gagliardi Urrutia, Frank Glaw, Juan M. Guayasamin, Monique Hölting, Martin Jansen, Philippe J. R. Kok, Axel Kwet, Rodrigo Lingnau, Mariana Lyra, Jiří Moravec, José P. Pombal, Fernando J. M. Rojas-Runjaic, Arne Schulze, J. Celsa Señaris, Mirco Solé, Miguel Trefaut Rodrigues, Evan Twomey, Celio F. B. Haddad, Miguel Vences, Jörn Köhler

**Affiliations:** 1 Division of Evolutionary Biology, Zoological Institute, Technical University of Braunschweig, Braunschweig, Germany; 2 Pós-graduação em Sistemática e Evolução, Centro de Biociências, Universidade Federal do Rio Grande do Norte, Campus Universitário Lagoa Nova, Natal, RN, Brasil; 3 Departamento de Ciencias Biológicas, Universidad de los Andes, Bogotá, Colombia; 4 Smithsonian Tropical Research Institute, Panamá, Republic of Panama; 5 Universidade de São Paulo, Instituto de Biociências, Departamento de Zoologia, São Paulo, Brasil; 6 Trier University, Biogeography Department, Trier, Germany; 7 CNRS-Guyane - USR3456, Immeuble Le Relais - 2, Cayenne, French Guiana; 8 Departamento de Zoologia, Instituto de Biociências, UNESP, Rio Claro, São Paulo, Brasil; Instituto de Investigación Biológica del Paraguay, Asunción, Paraguay; 9 Museo Nacional de Ciencias Naturales, Madrid, Spain; 10 Museum of Zoology, Senckenberg Natural History Collections Dresden, Dresden, Germany; 11 Peruvian Center for Biodiversity and Conservation (PCRC), Nanay, Iquitos, Peru; 12 Zoologische Staatssammlung München, München, Germany; 13 Universidad Tecnológica Indoamérica, Centro de Investigación de la Biodiversidad y el Cambio Climático (BioCamp), Cotocollao, Quito, Ecuador; 14 Senckenberg Gesellschaft für Naturforschung, Frankfurt am Main, Germany; 15 Amphibian Evolution Lab, Department of Biology, Vrije Universiteit Brussel, Brussels, Belgium; 16 German Herpetological Society (DGHT), Mannheim, Germany; 17 Universidade Tecnológica Federal do Paraná, Francisco Beltrão, PR, Brasil; 18 Departamento de Zoologia, Instituto de Biociências, UNESP, Rio Claro, São Paulo, Brasil; 19 Department of Zoology, National Museum, Prague, Czech Republic; 20 Departamento de Vertebrados, Museu Nacional, Universidade Federal do Rio de Janeiro, Rio de Janeiro, Brazil; 21 Fundación La Salle de Ciencias Naturales, Museo de Historia Natural La Salle, Caracas, Venezuela; 22 Hessisches Landesmuseum Darmstadt, Department of Zoology, Darmstadt, Germany; 23 Laboratorio de Ecología y Genética de Poblaciones, Centro de Ecología, Instituto Venezolano de Investigaciones Científicas, Caracas, Venezuela; 24 Universidade Estadual de Santa Cruz, Departamento de Ciências Biológicas, Rodovia Ilhéus-Itabuna, Bahia, Brasil; 25 Universidade de São Paulo, Instituto de Biociências, Departamento de Zoologia, São Paulo, Brasil; 26 Department of Biology, East Carolina University, Greenville, North Carolina, United States of America; Australian Museum, Australia

## Abstract

Species distributed across vast continental areas and across major biomes provide unique model systems for studies of biotic diversification, yet also constitute daunting financial, logistic and political challenges for data collection across such regions. The tree frog *Dendropsophus minutus* (Anura: Hylidae) is a nominal species, continentally distributed in South America, that may represent a complex of multiple species, each with a more limited distribution. To understand the spatial pattern of molecular diversity throughout the range of this species complex, we obtained DNA sequence data from two mitochondrial genes, cytochrome oxidase I (COI) and the 16S rhibosomal gene (16S) for 407 samples of *D. minutus* and closely related species distributed across eleven countries, effectively comprising the entire range of the group. We performed phylogenetic and spatially explicit phylogeographic analyses to assess the genetic structure of lineages and infer ancestral areas. We found 43 statistically supported, deep mitochondrial lineages, several of which may represent currently unrecognized distinct species. One major clade, containing 25 divergent lineages, includes samples from the type locality of *D. minutus*. We defined that clade as the *D. minutus* complex. The remaining lineages together with the *D. minutus* complex constitute the *D. minutus* species group. Historical analyses support an Amazonian origin for the *D. minutus* species group with a subsequent dispersal to eastern Brazil where the *D. minutus* complex originated. According to our dataset, a total of eight mtDNA lineages have ranges >100,000 km^2^. One of them occupies an area of almost one million km^2^ encompassing multiple biomes. Our results, at a spatial scale and resolution unprecedented for a Neotropical vertebrate, confirm that widespread amphibian species occur in lowland South America, yet at the same time a large proportion of cryptic diversity still remains to be discovered.

## Introduction

The application of molecular methods has expedited tremendously the discovery and characterization of global biological diversity [1]. This is particularly true for amphibians, where the rate of species descriptions has accelerated enormously in the past 20 years [2]–[7]. Integrative approaches that combine multiple lines of evidence have allowed taxonomists to define and name many of these evolutionary independent lineages as proper species [8]–[11]. The improved delimitation of species diversity, transforming one widely distributed species into several species, each with a smaller range, in many cases has notable impact on conservation. For instance, the International Union for Conservation of Nature (IUCN) status of certain populations may change from ‘Least Concern’ to one of the various threat categories or simply ‘Data Deficient’ [12]–[14].

Cryptic genetic diversity is now so commonly reported in molecular studies of amphibian species that the existence of nominally widespread tropical species has been called into question [15], [16]. However, supposedly widespread species occurring across multiple biomes and countries are rarely comprehensively sampled across their complete geographic range in screenings of genetic diversity [5], [6] or phylogeographic studies [17]–[21]. Sampling of species from across vast continental areas and across political borders is often handicapped by financial, logistic and political factors.

In the Neotropics, nominal taxa such as *Rhinella margaritifera* (Bufonidae), *Leptodactylus fuscus* (Leptodactylidae), and *Scinax ruber* (Hylidae) are prominent examples of anuran species once considered to occur across nearly the entire tropical lowlands of South America. Evidence has accumulated that many such putatively widespread species could in fact be complexes of cryptic taxa (e.g. [20], [22]). However, given limited genetic sampling and the difficulty in reviewing material from all countries hosting populations, their relationships and systematics remain in many cases as unclear as they were decades ago [23], [24].

A further example of a putatively widespread Neotropical amphibian species is *Dendropsophus minutus* (Peters, 1872), a small hylid frog of 21–28 mm snout-vent length, distributed in Cis-Andean South America, including the Andean slopes, the Amazon Basin, the Guiana Shield, down to the Atlantic Forests of southeastern Brazil, with an elevational record from near sea level up to 2,000 m [25]. Variation in coloration, osteology, advertisement calls and larval morphology [6], [26]–[29], along with molecular data from limited parts of the species' distribution [21] suggest that the nominal *D. minutus* might represent a species complex. However, the sheer size of its supposed geographical range along with nomenclatural and taxonomic complexity (six junior synonyms, [25]) and unresolved relationships in the *D. minutus* species group [30] have so far made these frogs inaccessible to revision.

In this case study, we use *D. minutus* to understand to what degree a tropical, small-sized anuran has the potential to be continentally widespread with limited genetic structure within its range, as expected for a single species. In addition to conservation concerns, this question has important implications for South American biogeography in general and amphibian systematics and evolution in particular. Evidence is accumulating that body size in amphibians has a positive correlation with range size [31], [32], but contrary to this trend many Holarctic amphibians occur with little genetic substructure across the vast ranges they colonized after the last glaciation, despite sometimes moderate to small body sizes (examples in [16]). Whether such patterns also exist across vast ranges in tropical regions, with their distinct historical climatic dynamics [33], is an open question. Deciphering possible cryptic diversity within the nominal *D. minutus* would also help inform conservation assessments which typically use species'geographic distributions as criteria for conservation status [13].

The present study is a multinational collaborative effort to sample nominal *D. minutus* across its entire range and at a spatial resolution unprecedented for a Neotropical vertebrate. Based on mitochondrial DNA sequences as a proxy for overall genetic diversity, we identify genealogical lineages currently subsumed within *D. minutus* and putative allies and assess their historical relationships and geographic ranges. Although there are some data on morphology and bioacoustics, we only partially discuss these here and refrain from making taxonomic decisions, but instead provide a roadmap for future integrative studies. Our focus, therefore, is on the biogeographical implications of the phylogeographic origins and evolutionary history of the *D. minutus* species group. We reveal here that this species complex exists as a mixture of both geographically widespread lineages and probable microendemic lineages.

## Methods

### Data collection and laboratory methods

No experiments were conducted using living animals. All field researches and collecting of specimens were approved by competent authorities, these being: Instituto Chico Mendes – ICMBio, Brazil, through collection permits granted to MG (21710-2), VGDO (19920), RL (26957-1), MTR (10126-1), CFBH (22511-1) and JPP (12600-2); Museo Nacional de Historia Natural – Colección Boliviana de Fauna, La Paz, Bolivia (permits: CBF CITE No. 02/2006 and No. 81/2007); DGB and the INRENA (granted permits to IDLR for collecting Bolivian and Peruvian material respectively); The Guyana Environmental Protection Agency and the Guyanese Ministry of Amerindian Affairs and Environmental Protection Agency of Guyana through research permit granted to PJRK's (180609 BR 112); Ministerio de Ambiente, Vivienda y Desarrollo Territorial of Colombia through research and collecting permit granted to AJC (No. 15 of 26 July 2010 and access to genetic resources permit No. 44); Ministerio Venezolano del Poder Popular para el Ambiente through permits granted to FJMRR and MHNLS (collection permit #4156, period 2009-2010; access to genetic resources permit #0076 of 22 February 2011). Samples from Ecuador were obtained under permits MAE-DPP-2011-0691 and 05-2011-Investigación-B-DPMS/MAE. Voucher specimens were euthanized using methods that do not require approval by an ethics committee. None of the species collected for this study is listed in the Convention on International Trade in Endangered Species of Wild Fauna and Flora – CITES (www.cites.org).

**Figure 1:**
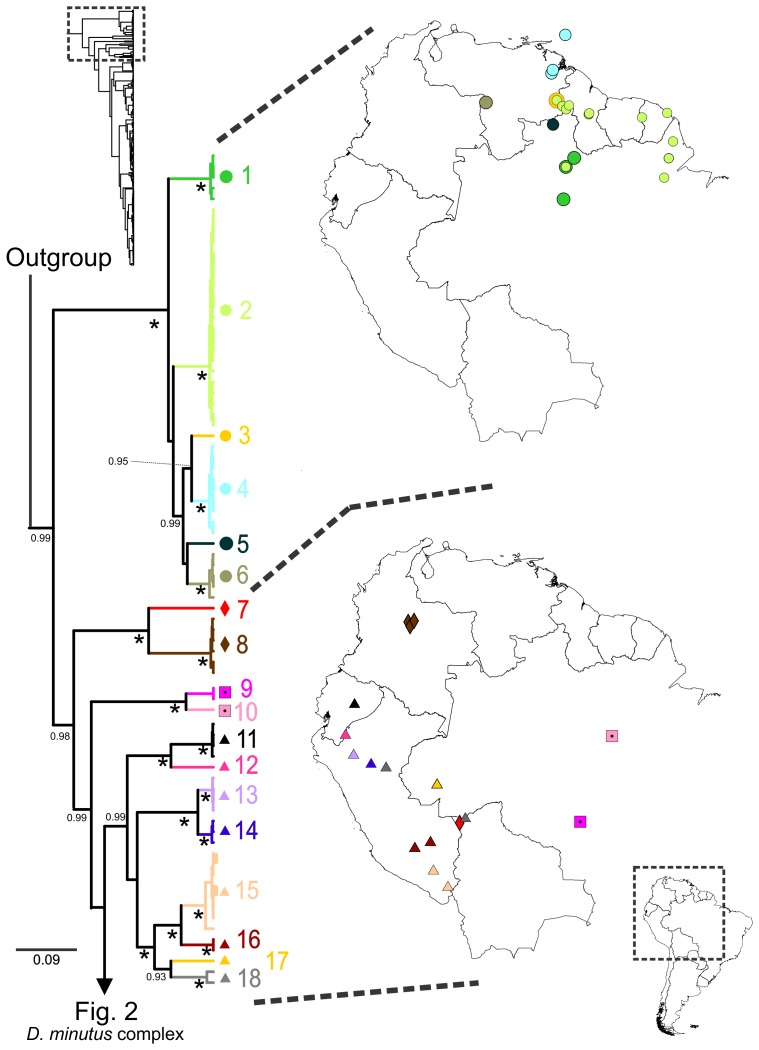
*Dendropsophus minutus* tree with lineage distribution part 1. 50% Maximum Clade Credibility tree and distribution maps of mtDNA lineages 1–18. Asterisks represent nodes with posterior probability equal to 1. Posterior probabilities lower than 0.9 are not shown.

**Figure 2:**
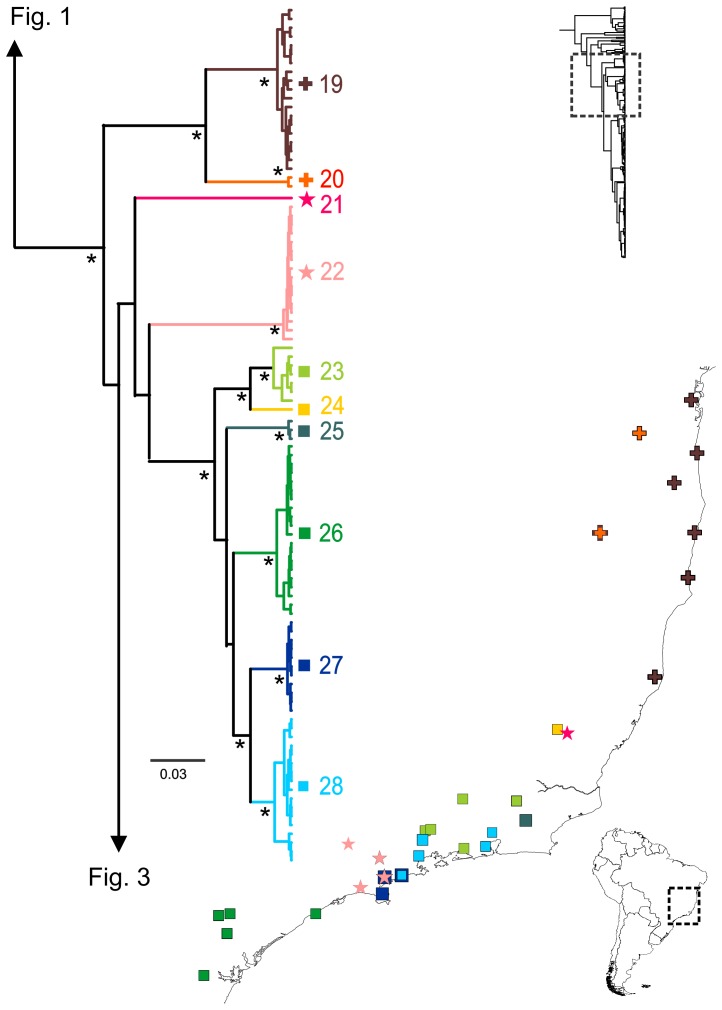
*Dendropsophus minutus* tree with lineage distribution part 2. 50% Maximum Clade Credibility tree and distribution maps of mtDNA lineages 19–28. Asterisks represent nodes with probability equals to 1. Probabilities lower than 0.9 are not shown.

**Figure 3:**
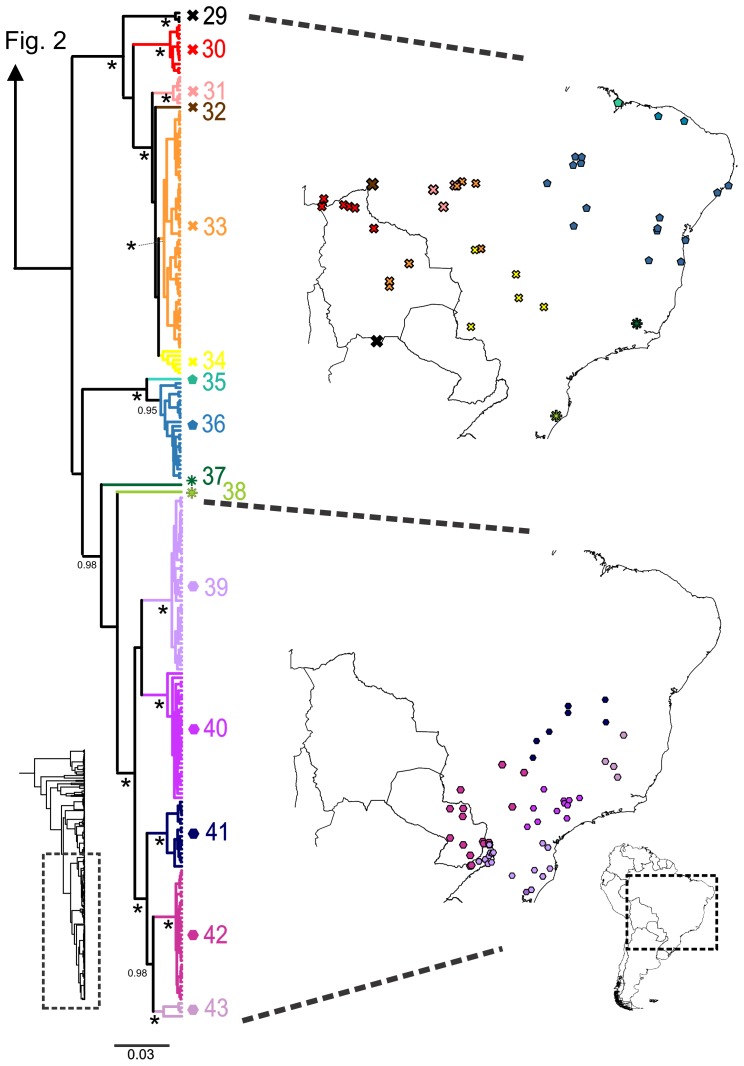
*Dendropsophus minutus* tree with lineage distribution part 3. 50% Maximum Clade Credibility tree and distribution maps of mtDNA lineages 29–43. Asterisks represent nodes with probability equals to 1. Probabilities lower than 0.9 are not shown.

We analyzed 407 samples of specimens identified as *Dendropsophus aperomeus*, *D. delarivai*, *D. minutus*, *D. stingi* or *D. xapuriensis* which we here consider together with *D. limai* (not included in our analysis), to constitute the *Dendropsophus minutus* species group (see Results, Figures 1–3 and Figures S1-2). The *D. minutus* species group was defined by Faivovich et al. (2005) [30] to comprise *D. delarivai*, *D. limai*, *D. minutus* and *D. xapuriensis*. These authors tentatively allocated *Dendropsophus aperomeus* to the *D. minimus* species group (in accordance with [34]). However, later molecular phylogenetic analyses suggested different positions for *D. aperomeus* [35]–[38]. *Dendropsophus stingi* has not been associated with any species group so far [30], but shares morphological characters with *D. minutus* [28], [39]. Because of their unsolved relationships, *D. aperomeus* and *D. stingi* were included in our study.

Genomic DNA was extracted in multiple laboratories using various routine methodologies. We used polymerase chain reaction and direct sequencing with PCR primers on automated Sanger sequencers to obtain DNA sequences of two mitochondrial gene fragments: the 16S ribosomal RNA gene and the 5′ portion of the cytochrome oxidase subunit I (COI) gene, the latter corresponding to the standard DNA barcode fragment [40]. See Supplementary Materials for detailed protocols and primers. Sequence alignment was performed using the MUSCLE algorithm [41] as implemented in the software MEGA version 5.0 [42]. 16S sequences were available for all samples (407), while COI sequences were only available for a subset of these (335). Gapped regions of the 16S alignment were treated as missing data. To fill the concatenated alignment missing COI sequences were also treated as missing data. All newly determined sequences were deposited in GenBank (accession numbers: KJ817824 - KJ817835, KJ833032 - KJ833585, KJ933533 - KJ933690, KJ940033 - KJ940049, see also Table S1 for detailed information of data collection).

### Substitution rate estimation and monophyly of the *Dendropsophus minutus* species group

To evaluate the monophyly of the *D. minutus* group and to estimate a substitution rate that could be used for calibrating the phylogeographic analysis (see below), we constructed a time-calibrated 16S gene tree with node constraints based on fossil and geographic evidence [30], [43], [44], comprising sequences of 216 species of hylids (Table S2), including all *Dendropsophus* species available from GenBank, plus 28 sequences representing the main clades of the *D. minutus* group. Regions of the 16S DNA sequence data that did not overlap our 16S alignment were excluded to ensure that the fragments used in the substitution rate estimation and in the phylogeographic analysis were the same. Additionally we included one sequence representing each main clade of *D. minutus* found in this study.

The calibrated tree was estimated using Bayesian Inference with posterior probabilities via a Markov Chain Monte Carlo (MCMC) sampling as implemented in the software BEAST version 1.7.2. We used a temporal calibration scheme similar to what was used in previous studies of hylids and other amphibians [44], [48]–[50], as follows. We constrained three nodes with uniform priors based on fossil evidence: 1) most recent common ancestor (MRCA) of *Acris* and *Pseudacris* to 15 million years (Ma) or older; 2) MRCA of *Hyla squirella* and *H. cinerea* to 15 Ma or older; and 3) MRCA of all *Hyla* as 33 Ma or older [51]. Additionally, we assumed the MRCA of Phyllomedusinae and Pelodryadinae to be related to the separation between South America and Australia [52], [53]. Therefore, we applied a fourth constraint to the respective MRCA of the two subfamilies using a normal prior with 40 Ma of mean and standard deviation of 6 Ma to allow a higher sampling probability around this value (quantiles: 5% = 30 Ma and 95% = 49.9 Ma) while avoiding hard boundaries. See Methods S1 for further information.

Substitution models were selected using the Akaike Information Criterion [45] in jModeltest version 0.1 [46] which suggested a GTR+I+Γ model for the 16S data set. A coalescent constant size tree prior was used with a lognormal uncorrelated relaxed clock model. The chain was run for 10^8^ steps, sampling every 10^4^ steps. We repeated the analysis five times to ensure convergence of posterior distributions. A maximum credibility clade tree was summarized using Tree Annotator version 1.7.2 provided in the BEAST 1.7.2 package. The first 20% of the trees were discarded as burn-in after empirical assessment of appropriate chain convergence and mixing with Tracer version 1.5 [47]. We used the median of the posterior density of the substitution rate (*ucld.mean*) obtained by this analysis as prior for phylogeographic analysis of the *D. minutus* complex. See Methods S1 for additional information.

### Gene tree inference, GMYC analysis and genetic distance calculations

We estimated an ultrametric mitochondrial gene tree using BEAST software, and all available 16S and COI sequences of the *D. minutus* group (1,068 bp concatenated alignment). As outgroups we included samples of *Dendropsophus nanus, D. bipunctatus* and *D. microcephalus*. We subdivided the data set into three partitions: (1) 16S, (2) third codon positions of COI, and (3) combined first and second positions of COI, and implemented a GTR+I+Γ model for each of the partitions, as suggested by the software PartitionFinder [54]. Bayesian inference of an ultrametric phylogenetic tree, using BEAST software, followed the method described above for the taxonomically more inclusive 16S data set.

The resulting ultrametric consensus tree was used as an input to estimate the number of statistically (and presumed evolutionarily) distinct lineages using the generalized mixed Yule-coalescent (GMYC) algorithm. Mitochondrial DNA sequence variation can provide a preliminary yet objective estimate of the number of phenetic clusters or evolutionary lineages represented in a given dataset [55], [56]. Of the many available algorithms [57], [58], we applied the GMYC algorithm which identifies clusters of mtDNA haplotypes by testing for a transition between fast coalescent rates within clusters relative to slower times to common ancestry among clusters, i.e., as described by a stochastic birth-only Yule model in forward time [59]. GMYC is expected to perform best under dense spatial sampling and limited migration [60], [61], two criteria which our study of the *D. minutus* species group would seem to meet (see below). Each statistically significant cluster identified by GMYC may correspond to a deep conspecific lineage or possibly an undescribed species, depending on support from other available taxonomic data [62]. The GMYC algorithm requires only an ultrametric tree as input, for which we used the concatenated 2-gene dataset (see above). We compared the likelihood of a single versus multiple transition threshold model via a *χ* ^2^ likelihood ratio test [63]. All GMYC calculations were performed using the ‘splits’ package, downloaded as ‘gmyc.pkg.0.9.6.R’ for the R statistical platform [64].

Using the clusters identified in the GMYC analysis, we calculated the uncorrected pairwise *p*-distances between these lineages and the mean *p*-distances within lineages using the 16S sequences only with pairwise deletion in the software MEGA5 [42]. Additionally, to determine the geographical extent of each lineage we used the occurrence data to generate minimum convex polygons representing the distribution range of each lineage observed in more than two localities. We then calculated the area (in km^2^) of each polygon using a Winkel-tripel projection in ArcGIS 10 software (ESRI, Redlands, CA).

### Phylogeographic analysis and connectivity surfaces

To reconstruct dispersal pathways within the *D. minutus* species group we applied a phylogeographic method that reconstructs geographical coordinates at the nodes of the genealogy using continuous trait reconstruction assuming a log-normal Relaxed Random Walk model implemented in BEAST software. The method estimates ancestral traits and topology simultaneously in a Bayesian framework, taking into account uncertainty of the topology [65]. For details of our implementation of this method, see Methods S1. Analyses assumed a coalescent prior with constant population size and an uncorrelated log-normal relaxed clock model [66], as well as an HKY+Γ model of substitution for each of the partitions used also in the gene tree estimation (see above). We ran three independent chains of 5×10^8^ iterations with different random seeds sampling every 50,000 steps. In order to calibrate the tree we used the substitution rate previously estimated for the 16S fragment using a wider time tree analysis of *Dendropsophus* (see above). Mixing of parameter sampling, effective sample size (ESS) and convergence were checked in Tracer 1.5. The resulted tree was summarized with Tree Annotator 1.7.2. The software SPREAD [67] was used to generate a *kml* (Keyhole Markup Language) file which was plotted in a Google Earth map (http://earth.google.com).

To obtain independent support for these dispersal pathways derived from phylogeographic analysis, we constructed conductance maps for the *D. minutus* species group based on Species Distribution Models (SDM) using the software Circuitscape version 3.5.8 [68]. This approach does not incorporate phylogeographic information but uses circuit theory to predict connectivity between localities or areas in heterogeneous landscapes. The algorithm explicitly incorporates effects of limited and irregular habitat extent accounting for multiple pathways and wider habitat bands connecting populations [69]. SDMs can be taken as possible conductive surfaces where highly suitable areas would have high conductance (or low resistance to dispersion) and low suitability areas would have low conductance (or high resistance to dispersion). We thus used a correlative niche model approach and derived a SDM to be used as a base layer for constructing conductivity surfaces.

As our data might represent multiple cryptic species or lineages, a SDM generated on the basis of all samples could be affected by the magnitude of the differences in climatic niches among the different lineages. To assess the extent of climatic niche diversification in the *D. minutus* group, we plotted the first two PC scores of a Principal Component Analysis (PCA) calculated from the values of 19 bioclimatic variables associated with each locality. Given the strong overlap of bioclimatic niches, especially of the majority of lineages 19–43 (Figure S3), i.e., the *D. minutus* complex, we combined these for constructing a single SDM. A robust test of niche conservatism between the lineages (as presented by [70]) was not applicable in our study because only a low number of localities was available for most lineages and because these tests often overestimate niche differentiation and are highly sensitive to sampling bias [71]. We furthermore projected the SDM to climatic scenarios representing past warm and cold extremes of the Late Pleistocene (Last Interglacial, 120 kyrBP, Last Glacial Maximum, 21 kyrBP). The SDM was constructed using six bioclimatic variables (see Methods S1) [72] using MaxEnt version 3.3.3a [73], [74]. The maps of the SDMs obtained for the present and past climate scenarios (see Figure S4) were then used as the input for Circuitscape along with the localities of the whole *D. minutus* group to calculate a conductance map between all pairs of sampled localities. The four resulting conductance maps were averaged. In addition, to highlight areas that under different models maintained high conductance, we applied a 25% quartile threshold to generate binary conductance maps, keeping the grids with higher values of conductance. The maps were then superimposed and spatially summed to highlight the areas of high sustained conductance (stable corridors). All geospatial processing was performed in ArcGis 10 (ESRI; Redlands, CA). See Methods S1 for detailed methodology.

## Results

The 16S tree containing all *Dendropsophus* for which sequences were available recovered the monophyly of the *D. minutus* species group (Figure S1). Within the group, the clade containing samples representing lineages 19–43 received a maximal posterior probability (1.0) and is defined here as the *D. minutus* complex (Figures 1–3, Figure S2B-D), given that lineage 25 contains samples from the type locality of *D. minutus* (Figures S2B). The substitution rate for the 16S fragment estimated from this analysis was 7.35×10^−3^/site/Ma [median of *ucld.mean* parameter (95% HPD = 6.1–8.7×10^−3^)], or 1.47% total divergence per Ma. The exclusion of the third temporal constraint involving the MRCA of the genus *Hyla* (Methods S1) did not change substantially the substitution rate estimate.

The 16S sequences had an average of 477 base pairs (bp) across individuals (standard deviation: 9.6) while the COI sequences had an average of 586 bp (standard deviation: 19.8). The alignment of all 16S sequences of the *D. minutus* species group contained 407, while the COI fragment contained 335 samples. The GMYC analysis on the concatenated data identified 43 entities excluding the outgroup samples, consisting of 31 clusters and 12 ‘singletons’, i.e., an entity consisting of a single concatenated mtDNA haplotype. The likelihood ratio test failed to reject the single-threshold GMYC model (χ^2^_ 9 d.f._ = 6.09, *P*-value = 0.7308), thus the following results are based on this simpler model, which tends to be more conservative in estimating number of statistically significant clusters.

Most of the mitochondrial lineages containing more than one sample received strong nodal support (Figures 1–3). The lineages splitting off from basal nodes of the tree (lineages 1–18) are distributed in the Guiana Shield, and in the Andean region of Peru, Ecuador and Colombia, with an eastern extralimital clade assembling disjunt localities in Mato Grosso and Pará (lineages 9–10; Figure 1).

The remaining lineages are in general more widely distributed in central and eastern South America (Figures 2–3). Lineages are largely allopatric but several cases of sympatry were observed (Figure S5). The uncorrected pairwise distances between lineages for the 16S gene ranged from 0.7 to 13%, while within-lineage *p*-distances ranged from 0.0 to 1.8% (Table S3).

**Figure 4:**
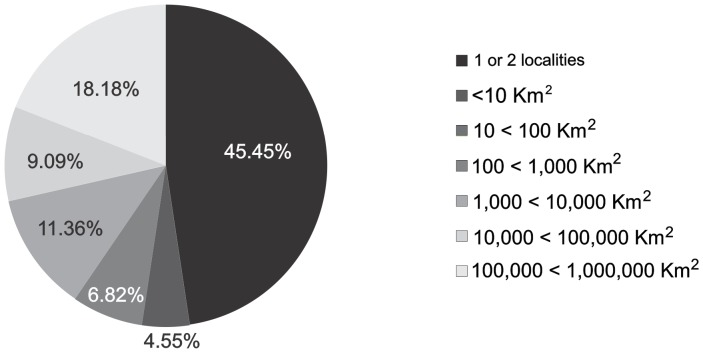
Comparative range size of lineages. Range size of lineages within the *Dendropsophus minutus* complex. Most lineages appear to be microendemic and are only recorded from one or two localities while eight lineages have ranges larger than 100,000 km^2^.

Most of the lineages (45%) were found in only one or two localities. Fifty per cent of the lineages were only found in areas smaller than 10 km^2^, and more than 70% have known ranges smaller than 10,000 km^2^. Eight out of the 43 lineages have a distribution larger than 100,000 km^2^ (Figure 4). Largest range sizes were found in northeastern Brazil (Caatinga domain; 997,262 km^2^, lineage 36), eastern Bolivia and western Brazil (Cerrado, Chaco and Dry Forest domains; 293,321 km^2^, lineage 33) and the Guiana Shield (269,741 km^2^, lineage 2).

**Figure 5:**
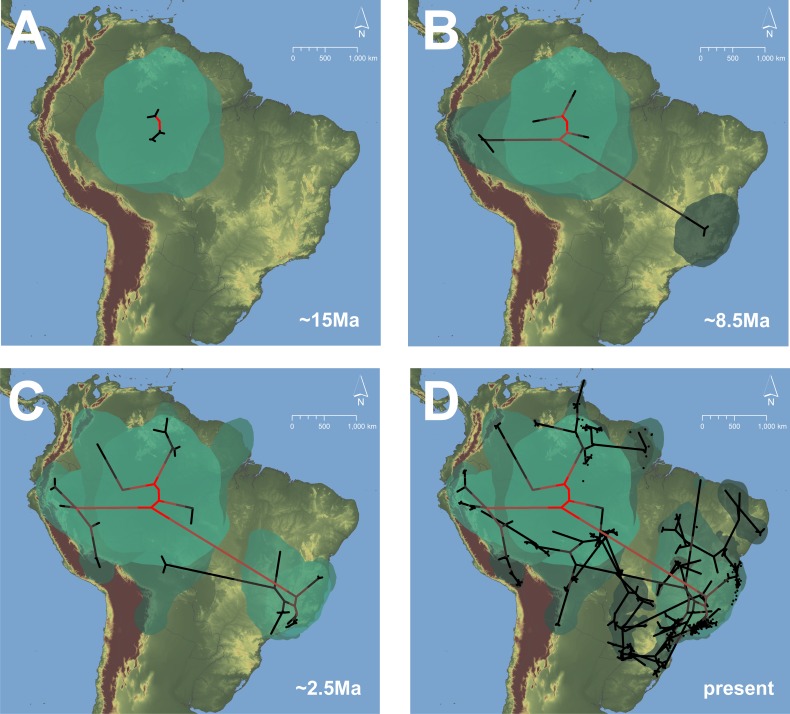
Phylogeographic reconstruction of *Dendropsophus minutus* group. Phylogeographical analysis of the *Dendropsophus minutus* group based on the 16S+COI mitochondrial dataset using a Relaxed Random Walk model for continuous trait reconstruction in Beast software. A) center of origin of the *D. minutus* group. B) Dispersal to west Amazonia, Guiana Shield, Andean region of Peru and eastern Brazil; polygon at the east represent the geographic origin of lineages representing the *D. minutu*s complex. C) Dispersal from east Brazil to lowland of Bolivia; further dispersal to Guiana Shield, Peruvian and Colombian areas. D) Recent dispersals to northeast and south Brazil, east Paraguay and Guiana shield. Green polygons and red branches indicate relatively older events while dark polygons and black branches indicate later events. Maps were generated using google earth (earth.google.com).

**Figure 6:**
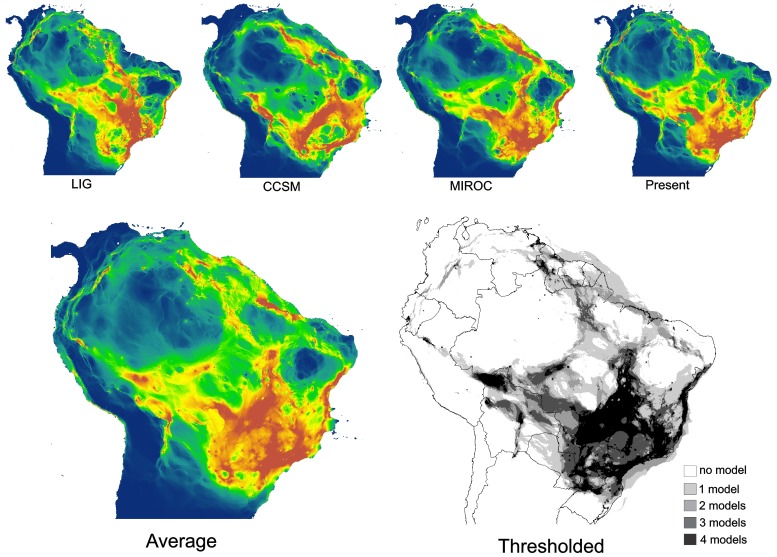
Estimated conductance maps for *Dendropsophus minuts* complex. Conductance maps constructed with the program Circuitscape. Conductances were estimated according to a spacial distribution modeling for the *D. minutus* complex projected to four different climatic scenarios. Conductance maps were averaged and thresholded to show stable dispersal corridors (see methods for details).

The phylogeographic analysis suggested an Amazonian origin for the *D. minutus* group (Figure 5A), with subsequent dispersal to the Atlantic Forest, Guiana shield and the Andean region (Figures 5B and 5C). From there, lineages then dispersed outward from the Atlantic Forest and Amazonia into the southern Atlantic Forest and eastern Paraguay, northeastern and central Brazil, and to the Guiana Shield (Figures 5C and 5D). The conductance analysis with Circuitscape software resulted in the most stable conductance areas along the Brazilian Atlantic coast, central Brazil, northeastern Argentina (Misiones region) and northern Bolivia. The analysis suggested three paths of connectivity between eastern Brazil and the Amazonian region: 1) across northeastern Brazil; 2) across central Brazil; and 3) across southern Brazil, the third being the most stable (Figure 6).

## Discussion

### Diversity in the *Dendropsophus minutus* species group

Given the numerous divergent lineages and high degree of differentiation revealed here, our molecular approach shows that more than one species could be hidden behind the name *Dendropsophus minutus*. At this stage, we refrain from any formal taxonomic action, as the clarification of the taxonomic status of the populations involved requires thorough integrative revision. In a few cases, there exists information on morphology and calls, but data are not sufficient to either support or reject species status for individual lineages, or allocate available names with certainty. In at least one example GMYC has been found to overestimate the number of species when compared to an integrative approach [75]. Thus, it is possible that several of the lineages identified by this method may not correspond to distinct species. Despite this qualification, some conclusions concerning species diversity can be derived from our results based on monophyly and GMYC results.

Concerning lineages in the *D. minutus* species group not belonging to the *D. minutus* complex (lineages 1–18; Figure 1), our analyses revealed multiple deeply differentiated lineages. Our results under the criteria of mitochondrial monophyly and statistically distinct genealogical lineage (GMYC) suggest recognition of the nominal taxa *D. aperomeus*, *D. delarivai*, *D. stingi* and *D. xapuriensis* as members of the *D. minutus* species group, whose monophyly has not previously been rigorously tested and the relationships among its species has not been studied. In two cases (*D. aperomeus*, *D. stingi*) the species were not previously allocated to the *D. minutus* species group [28], [30], [34], [39], [76]. Kaplan (1994) [28] acknowledged that *D. stingi* was morphologically similar to *D. minutus*, while Köhler and Lötters (2001) [39] pointed to similarities of *D. aperomeus* and *D. delarivai* (the latter species at that time being tentatively allocated to the *D. minutus* group based on phenetic similarities), and Wiens et al. (2005) [35] found *D. aperomeus* to be related to *D. minutus*. The relationships of *D. aperomeus* and *D. minutus* also remained unresolved in the analysis of Pyron and Wiens (2011) [37].

Among the *D. minutus* species group members external to the *D. minutus* complex, lineages 1–6 are Guianan, while 7–18 are primarily distributed along the Andean foothills, and all show well-pronounced molecular differentiation and divergence. Among lineages 1–6, there is moderate genetic differentiation. Considering mitochondrial reciprocal monophyly and GMYC results as criteria, and being taxonomically conservative, one available name, *Hyla goughi* Boulenger, 1911 (type locality: Trinidad), should likely be removed from the synonymy of *D. minutus* and allocated to populations comprised by all or some of lineages 1–6. As a conservative estimate, we hypothesize that lineages 7–18 comprise seven distinct species, *i.e.*, five named taxa and two undescribed species (lineages 9+10 and 11+12).

Within the *D. minutus* complex (i.e., *D. minutus* sensu lato), GMYC analyses revealed 25 divergent clades (lineages 19–43 in Figures 2 and 3, Figure S2B-D). In several cases, these lineages occur in sympatry (e.g., lineages 19 and 20; 19 and 36; 22 and 27; 27 and 28; 34 and 42). In one case (lineages 30 and 33; Figure 3) genetic differentiation between allopatric lineages is concordant with consistant differences in larval morphology (A. Schulze unpubl. Data, [6]), suggesting species differentiation. However, larval morphology of *D. minutus* has not yet been explored globally across its range. There are some descriptions of external morphology mainly based on single (or closely located) populations (e.g. [77], [78]–[84]) and one description of internal larval buccal morphology [85]. While larval morphology may offer an alternative source of abundant taxonomic characters [86], [87] it also can be highly plastic, suggesting that more data need to be collected and coded with care (e.g. [88], [89]).

Advertisement calls among populations which, according to our analyses, would represent distinct lineages, were in some cases shown to be very similar or even identical [26], [90]. The certain assignment of calls to any mitochondrial lineage may require sequencing of voucher specimens having recorded calls. On the other hand, call differences have been found among some lineages within *D. minutus* sensu lato identified here through molecular analyses [6], thus potentially providing further support for the presence of additional cryptic species.

Our analysis included topotypic material that may correspond to some of the available names currently regarded as synonyms of *D. minutus*. If additional integrative taxonomic studies are able to link unique diagnostic traits to our mtDNA clades, the following synonyms may require revalidation: *Hyla pallens* Lutz, 1925 (lineage 28), *H. velata* Cope, 1887 (lineage 33), and *H. bivittata* Boulenger, 1888 (lineage 39). On the other hand, *H. suturata* Miranda-Ribeiro, 1926 (lineage 28), and *H. emrichi* Mertens, 1927 (lineage 39) likely represent junior synonyms of *H. pallens* and *H. bivittata*, respectively. Samples from the type locality of *Hyla minuta* Peters, 1872 form a distinct clade (lineage 25) and do not cluster with samples from any other locality studied here (see Figures S2B-D). Results of the GMYC analyses should be interpreted with caution, however, as genealogical clusters identified by this method may correspond to species but may also simply be conspecific phylogeographic lineages [75].

At some localities our analyses revealed up to three independent lineages of the *D. minutus* species group occurring in sympatry or at least in very close proximity (e.g., lineages 21, 24, 37; lineages 22, 27, 28; lineages 7, 18, 30; lineages 2, 3, 5; Figures 2 and 3; Figure S5). Several of the sympatric yet highly divergent lineages are found in or close to areas recognized as Quaternary refugia (southern Bahia and southeastern São Paulo) [91], [92]. Phenotypically or genetically distinct groups that are able to maintain their genetic integrity in sympatry may be interpreted as biological species [93]. However, divergent mtDNA lineages in sympatry could also be the product of recent secondary contact between previously isolated yet conspecific (and freely interbreeding) populations [94]. Therefore, we prefer to interpret these sympatric entities as Deep Conspecific Lineages pending additional evidence for possible species status [7], [95].

### Biogeographic origins of the *Dendropsophus minutus* species group

The region of likely origin of the *D. minutus* group as suggested by the mitochondrial history (Figure 5A) corresponds to the Amazon Basin. The estimated time frame of coalescence of all mtDNA lineages at 18.0 Ma (95% HPD, 14.6–21.4 Ma) falls in the Early to Middle Miocene, a period following the first peak of mountain building in the Andes (∼23 Ma) and when, according to Hoorn et al. (2010) [33], western Amazonia was characterized by a large wetland formation of shallow lakes and swamps known as the Lake Pebas System (11–17 Ma). This was apparently an important period for the diversification of the Neotropical fauna and flora in the Amazonian region [33], [96], [97]. For instance, this period coincides with the diversification of other anurans such as the *Allobates trilineatus* complex 14.0–15.0 Ma [98], the *Rhinella marina* complex 10.7–17.2 Ma [99], the genus *Phyzelaphryne* 9.0–18 Ma [100] and the Amazonian *Adelophryne* clade 13.0–24.0 Ma [100]; as well as with the diversification of Amazonian gymnophthalmids of the genus *Leposoma* around 13.9 Ma [101].

The phylogeographic reconstruction of the *D. minutus* group suggests dispersal to the Andes and the Guiana Shield after its initial diversification between 8.0–11.0 Ma (Figures 5A and B), with additional recent dispersals (Figures 5C and D). This is in remarkable concordance with the pattern observed in dendrobatid frogs for which dispersals from the Amazon Basin into the same regions were reconstructed at 8.8–10.8 Ma, followed by another more recent wave 0.7–6.0 Ma [98]. Concomitant with the first dispersal wave there was a first long distance dispersal of *D. minutus* from the Amazonia to the Atlantic Forest.

An old relationship between Amazonia and an Atlantic Forest area at southeastern Bahia has long been proposed (the “Hiléia Bahiana” [102]). Independent molecular-based studies have recovered similar relationships (e.g. [103]) and studies with amphibians have provided additional evidence for this scenario [100], [104]–[107]. Some authors proposed that climatic changes in the Eocene, leading to the formation of a diagonal band of open and dry biomes (currently Caatinga, Cerrado and Chaco biomes) separating the Atlantic Forest from Amazonia, likely caused ancient vicariance of forest dwelling amphibians [103], [105], [106]. Accordingly, only generalist species that tolerate both dry and humid conditions could disperse across this potential environmental barrier, and therefore would be distributed in both wet-forest biomes [100]. On the other hand, evidence of Miocene dispersal of forest-dwelling frogs suggests the possibility of relatively more recent forested connections between Atlantic Forest and Amazonia [100], [107]. Our analysis recovers a Late Miocene dispersal of *D. minutus* from Amazonia to eastern Brazil (8–13 Ma) (Figures 5B, C and D). However, frogs of the group are currently found in a variety of habitats, including savannas and deciduous forest. Thus, this first dispersal event could have occurred without the necessity of dense forest continuity and may be unrelated to ancient dispersal of forest dweling species.

### Diversification and dispersal within the *Dendropsophus minutus* complex

The polygon corresponding to the reconstructed area of origin of the *D. minutus* complex encompasses potentially climatically stable areas within the Atlantic Forest [108]. From here, the complex further diversified. Within the Atlantic Forest, sympatry of lineages was observed within or close to Pleistocene refugia (lineages 19 and 20; lineages 21 and 24; lineages 22, 23, 25, 26, 27 and 28; Figure 2) suggesting that these stable areas [91], [92], [108] might be related to the persistence of deeply divergent lineages. The inferred history of lineage diversification further recovers two discontinuities also found in other vertebrates, possibly representing suture zones [109]. The first of these is located at the southern border of the state of São Paulo, Brazil, represented by the southern limit of the distribution of lineages 26 and 40, and the northern limit of lineage 39, a discontinuity that has also been observed in pit vipers and toads [92], [110], [111]. The second possible suture zone is in the state of Espirito Santo, Brazil, represented by the northern limit of lineages 21, 23 and 24, and the southern limit of lineage 19 (Figs. 2–3; Fugure S6), coinciding with genetic breaks observed in toads, geckos, foxes and woodcreepers [92], [112]–[114].

After the initial diversification of the *D. minutus* complex in the Atlantic Forest, our phylogeographic inference suggests subsequent dispersal to other areas of eastern South America, central Brazil and Amazonia, supporting a southern dispersal route between Amazonia and the Atlantic Forest (Figures 5C and D). Distribution patterns of different vertebrate groups, as well as climatic and floristic evidence, suggest recent dispersal corridors between these forested areas [103], [107], [114]–[116]. On the other hand, the phylogeography of tropical rattlesnakes adapted to dry biomes suggests past connectivity between open area formations that are currently isolated [117], [118]. These results combined with paleoclimatic models of the Atlantic Forest and Cerrado [108], [119] support the past existence of a dynamic interplay of dispersal corridors and temporary barriers across the South American lowlands. Our conductance analysis that took into account contrasting climatic scenarios suggests the existence of stable stretches of favorable habitat for *D. minutus* along northern and particularly southern Amazonia during the Pleistocene, which would have allowed recent dispersal between forested areas of South America, corroborating the phylogeographic results (Figure 6).

The stable habitat corridor inferred along southern Amazonia (Figure 6) was proposed previously as the main floristic connection between the Atlantic Forest and Amazonia [120] and phylogeographic analyses of some species of small mammals corroborate this hypothesis [103]. Moreover, the palynological record from different continents of the southern hemisphere suggest the existence of a band of moisture at this latitude (∼23°S) during the Last Glacial Maximum (LGM) [121], which raises the possibility that this same corridor may have persisted even earlier. The presence of a mosaic of forest and open formations, and higher moisture, could have generated conditions necessary for the existence of a Pleistocenic southern dispersal route for *D. minutus*.

### Widespread amphibian species in the Neotropics

Data presented herein provide conclusive evidence for a strong genetic subdivision of the nominal species *Dendropsophus minutus* as currently understood. Current taxonomy conservatively assumes a putatively widespread species encompassing a vast area of South America (from approximately latitude 11.0°N to 35.0°S), distributed across several biomes. Our results, however, reveal high genetic diversity within *D. minutus* that would suggest the existence of numerous distinct species, leading to an important increase in number of species. If this hypothesis is confirmed through further studies, the existence of an increased number of species with decreased range sizes would have important consequences for the definition of centers of endemism and for assessing conservation status.

Despite revealing a substantial amount of cryptic genetic diversity within *D. minutus* sensu lato, our results also confirm the existence of widespread Neotropical species of anurans. While we cannot yet confirm which of the mitochondrial lineages within the *D. minutus* complex will merit a status as separate species, we can inversely conclude that in most cases, all samples assigned to one mtDNA lineage should be conspecific. Although cases of distinct amphibian species with low mtDNA differentiation exist (e.g. [86]) and phenomena of mtDNA introgression can potentially blur species identities (e.g. [122], [123]), such cases remain exceptional. Therefore, these factors are unlikely to substantially affect our calculation of range sizes according to which a total of eight mtDNA lineages have ranges >100,000 km^2^ (lineages 2, 19, 33, 34, 36, 39 41, 42). In the most inflationist taxonomic scenario, with each of these lineages representing separate species, our dataset still provides evidence for a species of lowland Neotropical amphibian (lineage 36) occupying an area of almost one million km^2^, encompassing multiple biomes across a distance of about 1,600 km between its two most distant populations.

The most widespread lineages within *D. minutus* sensu lato have distributions restricted to or centered in Brazil and occur within rather open habitats, while lineage 2 of the *D. minutus* group (with a range of almost 270,000 km^2^) occurs in rainforest. Various of the lineages known from only few or single sites (e.g., lineages 8, 11, 12, 13, 14, 15, 16) occur in the Andean foothills or on mountain slopes. Nevertheless, in the Andean area, a higher sampling density is needed before it can be concluded with certainty that those lineages are restricted to small ranges. Hence, the distribution of mitochondrial lineages in the *D. minutus* group indicates that in open lowland areas of South America, small-sized species of anurans can be widespread.

### A plea for collaboration in taxonomy and large scale phylogeography

Whether a researcher is interested in taxonomic or biogeographic questions concerning widespread species groups and complexes, this study points to several advantages of analysing spatialy dense and geographically complete datasets. Large-scale analyses are of course pivotal to understanding processes at a continental level. Patterns may be misinterpreted when looking at a limited geographical area and/or limited sample size. Widespread groups suchs as *D. minutus* group are suitable for continental analyses, but they are often associated with taxonomic difficulties because of the lack of comprehensive datasets. Also, certain biogeographic analyses, like those performed here, damand extensive data. Through collaboration we can increase the efficiency of data collection, thus overcoming the problem of incomplete datasets.

The taxonomic and conservation crisis we are currently facing, where some species will be extinct before the community becomes aware of their existence [124], underscores the urgency and importance of collaborative work. Species extinction represents the loss of the genealogical and biogeographic information embedded in its evolutionary history. Broad collaboration among scientists is necessary to rapidly tackle taxonomic and biogeographic questions. As in other areas of science, we feel that in taxonomy and biogeography the establishment of multi-investigator, multi-institutional and multi-national consortia dealing with widely distributed and taxonomically convoluted groups will improve quality and speed of taxonomic revision, consequently improving our understanding of biodiversity patterns, the evolutionary processes that generated them, and the conservation status of tropical organisms.

## Supporting Information

Figure S1 **16S genealogy of the genus** ***Dendropsophus***: partial view of the 50% Maximum Clade Credibility tree derived from Bayesian phylogenetic inference of 216 mitochondrial 16S sequences of Hylidae species plus 28 exemplars of the *D. minutus* group, that was performed for the substitution rate estimations using the program BEAST 1.7.2. The *Dendropsophus minutus* group is highlighted by the dashed line and the *Dendropsophus minutus* complex by the grey box. Node numbers indicate posterior probabilities which are only shown when higher than 0.8. Numbers between brakets indicate lineage number acording with the GMYC results or GenBank accession numbers.
(DOCX)

Figure S2 **A.** ***Dendropsophus minutus*** **tree with samples names and annotations Part 1.** 50% Maximum Clade Credibility tree, lineages 1–18. Asterisks represent nodes with probability equals to 1. Probabilities lower than 0.9 are not shown. Annotations refer to samples of particular interest, mainly samples collected at or close to the type locality of certain nominal taxa. **B.** ***Dendropsophus minutus*** **tree with samples names and annotations Part 2.** 50% Maximum Clade Credibility tree, lineages 19–28. Asterisks represent nodes with probability equals to 1. Probabilities lower than 0.9 are not shown. Annotations refer to samples of particular interest, mainly samples collected at or close to the type locality of certain nominal taxa. **C**. ***Dendropsophus minutus*** **tree with samples names and annotations Part 3.** 50% Maximum Clade Credibility tree, lineages 29–36. Asterisks represent nodes with probability equals to 1. Probabilities lower than 0.9 are not shown. Annotations refer to samples of particular interest, mainly samples collected at or close to the type locality of certain nominal taxa. **D.** ***Dendropsophus minutus*** **tree with samples names and annotations Part 4.** 50% Maximum Clade Credibility tree, lineages 37–43. Asterisks represent nodes with probability equals to 1. Probabilities lower than 0.9 are not shown. Annotations refer to samples of particular interest, mainly samples collected at or close to the type locality of certain nominal taxa.
(DOCX)

Figure S3 **PCA of climatic variables.** PCA plots showing the first two principal components separately for lineages 1–18 (left) and 19–43 (right). Symbols and colours match those in Figs. 1–3. Loadings of the first two principal components were as follows (separated by colon): annual mean temperature 0.324, 0; mean monthly temperature range −0.122, 0.274; isothermality 0.192, 0; temperature seasonality −0.245, −0.13; maximum temperature warmest month 0.269, 0; minimum temperature coldest month 0.336, 0; temperature annual range −0.214, 0.123; mean temperature wettest quarter 0.274, 0; mean temperature driest quarter 0.336, 0; mean temperature warmest quarter 0.282, 0; mean temperature coldest quarter 0.339, 0; annual precipitation 0.187, −0.292; precipitation wettest month 0.226, 0; precipitation driest month 0, −0.477; precipitation seasonality 0, 0.421; precipitation wettest quarter 0.224, 0; precipitation driest quarter 0, −0.482; precipitation warmest quarter −0.113, −0.184; precipitation coldest quarter 0.132, −0.331. Main result of this analysis: the first two principal components (PCs) accounted for 64% of the climatic variance, with highest loadings for temperature variables along PC 1 (minimum temperature coldest month, mean temperature driest quarter, mean temperature coldest quarter) and precipitation variables along PC 2 (precipitation driest month, precipitation seasonality, precipitation of driest quarter). The PCA suggests that within the *D. minutus* group, the climatic niches (i.e., 19 bioclimatic temperature and precipitation dimensions) are rather similar, even when the two main groups are compared (i.e. lineages 1–18 vs. 19–43). Some lineages from the periphery of the known geographic distribution of the group, including lineages 1–18, are weakly separated (lineages 9, 11–13 15, 16).
(DOCX)

Figure S4 **Spatial distribution models used as resistance layers in the Circuit Scape analysis.**
(DOCX)

Figure S5 **Distribution of all mitochondrial lineages.** Map showing the distribution of all mitochondrial lineages in the *Dendropsophus minutus* group as revealed by this study. Symbols refer to those used in Figs. 1–3.
(DOCX)

Table S1 **Complete data collection with genbank accession numbers, voucher numbers and locality information.**
(CSV)

Table S2 **Species used in the substitution rate estimation with respective Genbank accession numbers.**
(DOCX)

Table S3 **Uncorrected pairwise** ***p*** **-distance among lineages and average within lineage** ***p*** **-distance.**
(XLSX)

Table S4 **List of primers used in this study with respective anealing temperatures.**
(DOCX)

Table S5 **Localities and coordinates used in the Spatial Distribution Modeling.**
(DOCX)

Methods S1 **Supplementary methods.**
(DOC)
