# Correction: High Levels of Diversity Uncovered in a Widespread Nominal Taxon: Continental Phylogeography of the Neotropical Tree Frog *Dendropsophus minutus*

**DOI:** 10.1371/journal.pone.0111829

**Published:** 2014-10-22

**Authors:** The PLOS ONE Staff

## Notice of Republication

*PLOS ONE* requires that all content is published under the Creative Commons Attribution License (CC BY). The original Figure 5 was not published under this license and as a result, we have removed the previous Figure 5 from this article and replaced it with an appropriate figure.
