# Supplementary figures and images for: High Levels of Diversity Uncovered in a Widespread Nominal Taxon: Continental Phylogeography of the Neotropical Tree Frog Dendropsophus minutus
Source: PLoS One. 2014 Sep 10;9(9):e103958. doi: 10.1371/journal.pone.0103958 (PMC4160190; doi:10.1371/journal.pone.0103958)

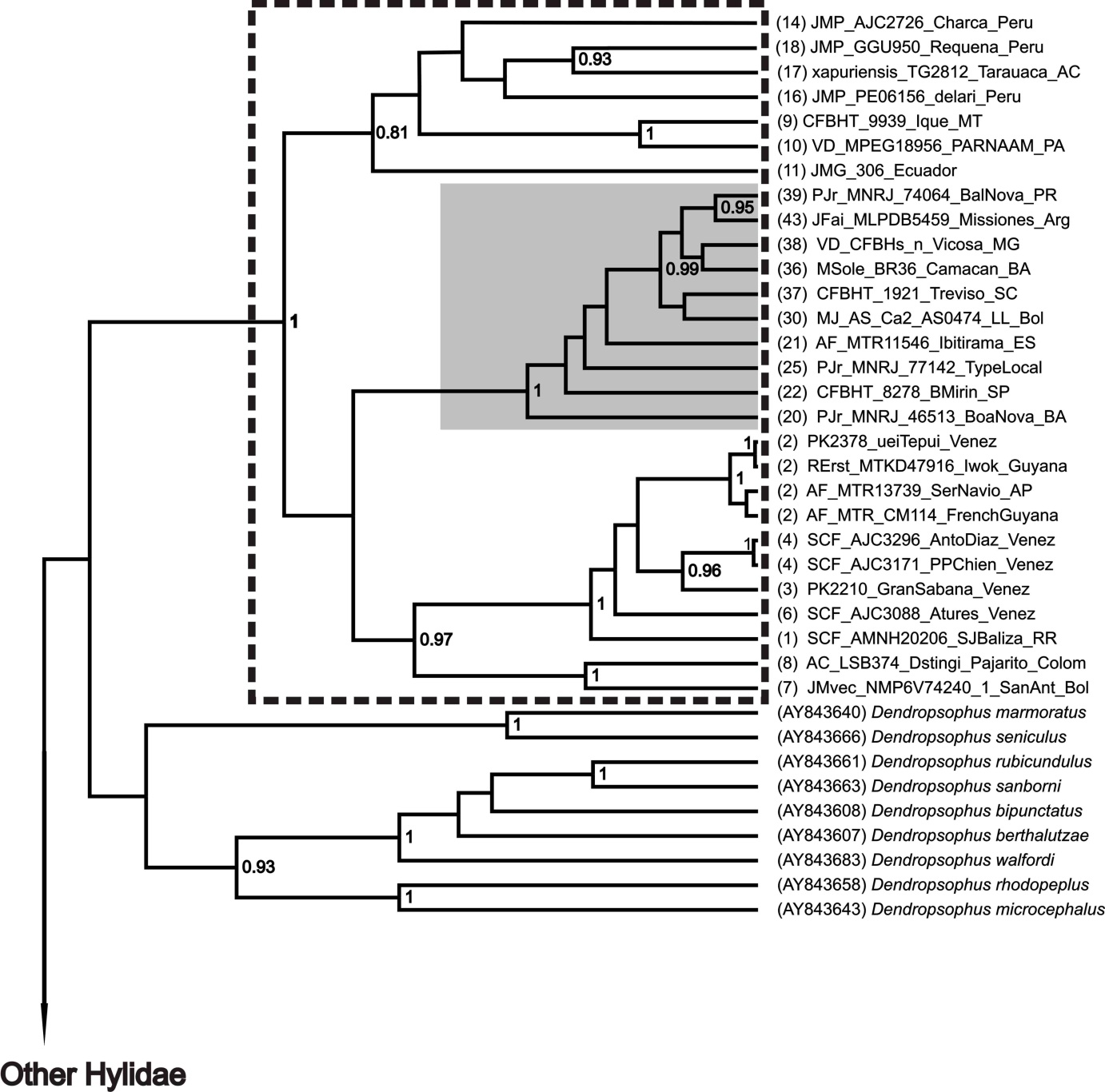

Supplement: Figure S1 — 16S genealogy of the genus Dendropsophus: partial view of the 50% Maximum Clade Credibility tree derived from Bayesian phylogenetic inference of 216 mitochondrial 16S sequences of Hylidae species plus 28 exemplars of the D. minutus group, that was performed for the substitution rate estimations using the program BEAST 1.7.2. The Dendropsophus minutus group is highlighted by the dashed line and the Dendropsophus minutus complex by the grey box. Node numbers indicate posterior probabilities which are only shown when higher than 0.8. Numbers between brakets indicate lineage number acording with the GMYC results or GenBank accession numbers. (DOCX) [file pone.0103958.s001.docx]

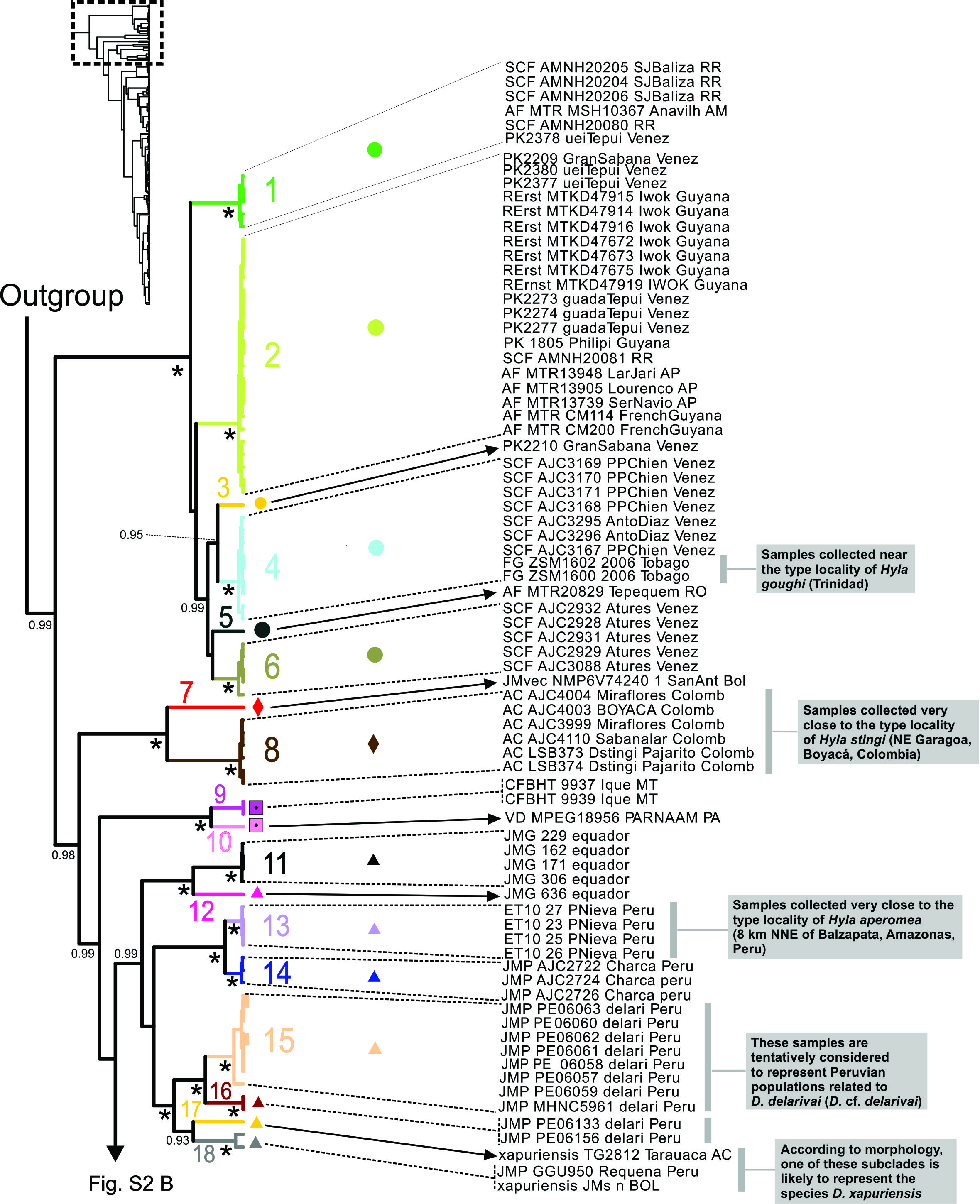


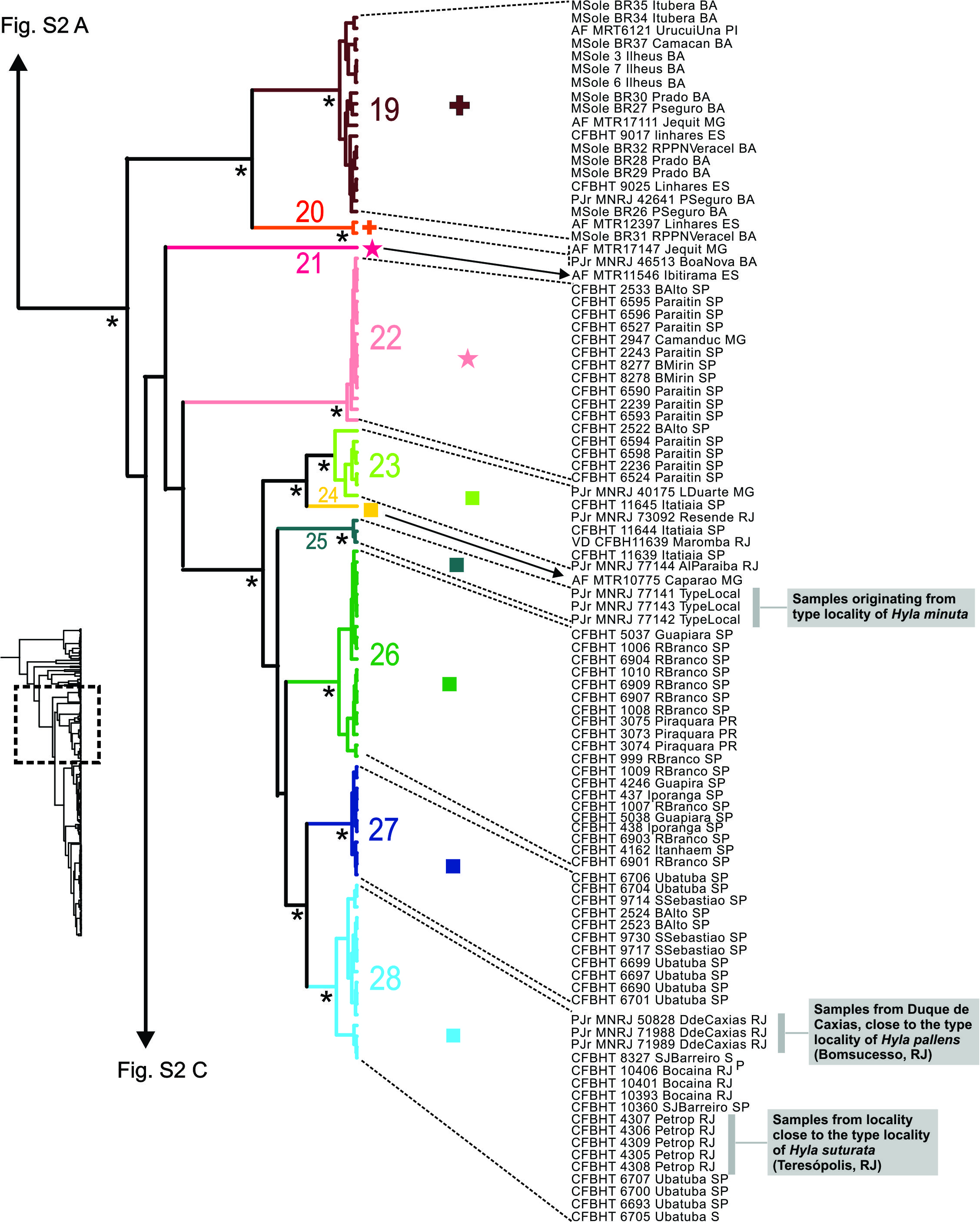


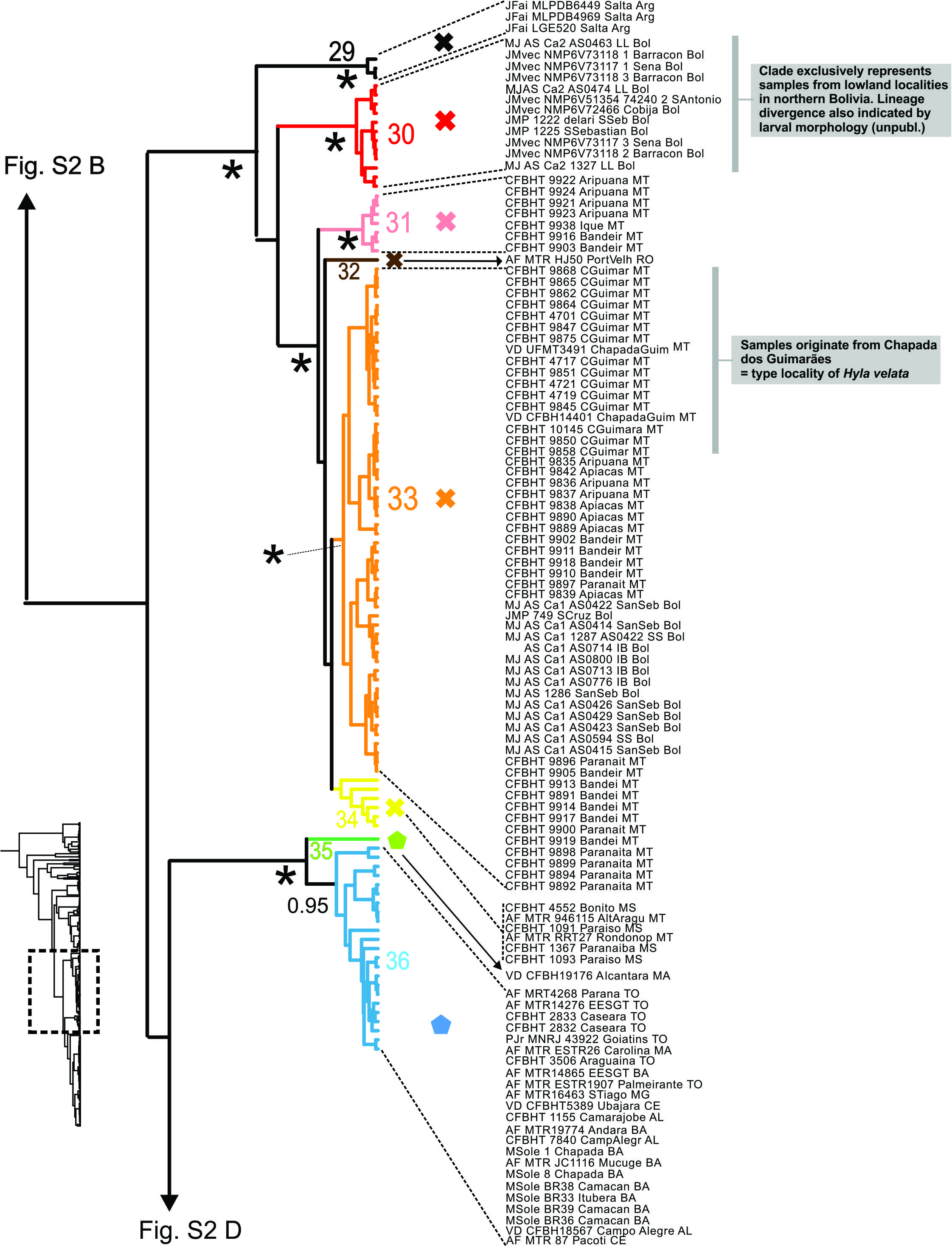


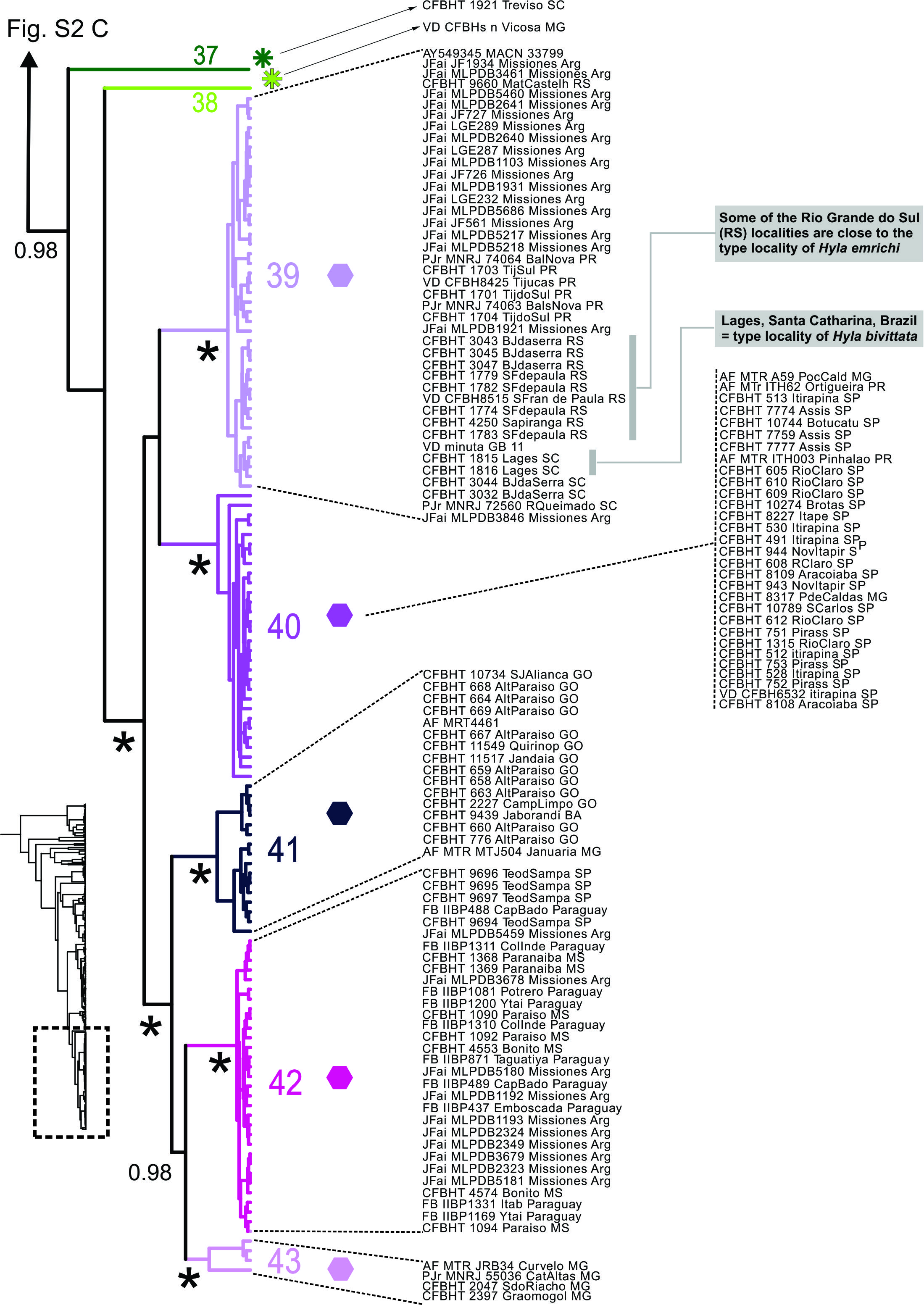

Supplement: Figure S2 — A. Dendropsophus minutus tree with samples names and annotations Part 1. 50% Maximum Clade Credibility tree, lineages 1–18. Asterisks represent nodes with probability equals to 1. Probabilities lower than 0.9 are not shown. Annotations refer to samples of particular interest, mainly samples collected at or close to the type locality of certain nominal taxa. B. Dendropsophus minutus tree with samples names and annotations Part 2. 50% Maximum Clade Credibility tree, lineages 19–28. Asterisks represent nodes with probability equals to 1. Probabilities lower than 0.9 are not shown. Annotations refer to samples of particular interest, mainly samples collected at or close to the type locality of certain nominal taxa. C. Dendropsophus minutus tree with samples names and annotations Part 3. 50% Maximum Clade Credibility tree, lineages 29–36. Asterisks represent nodes with probability equals to 1. Probabilities lower than 0.9 are not shown. Annotations refer to samples of particular interest, mainly samples collected at or close to the type locality of certain nominal taxa. D. Dendropsophus minutus tree with samples names and annotations Part 4. 50% Maximum Clade Credibility tree, lineages 37–43. Asterisks represent nodes with probability equals to 1. Probabilities lower than 0.9 are not shown. Annotations refer to samples of particular interest, mainly samples collected at or close to the type locality of certain nominal taxa. (DOCX) [file pone.0103958.s002.docx]

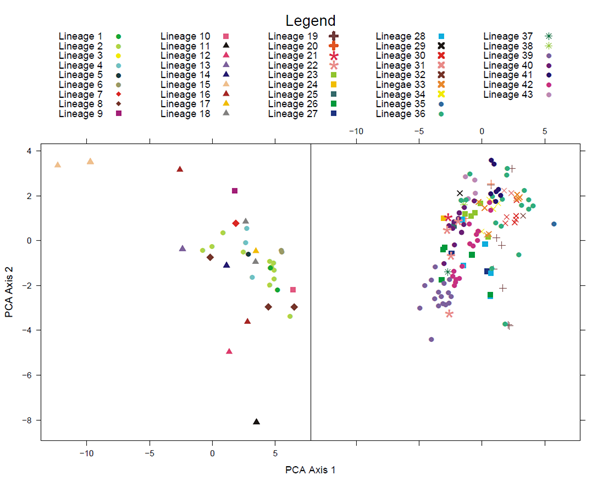

Supplement: Figure S3 — PCA of climatic variables. PCA plots showing the first two principal components separately for lineages 1–18 (left) and 19–43 (right). Symbols and colours match those in Figs. 1–3. Loadings of the first two principal components were as follows (separated by colon): annual mean temperature 0.324, 0; mean monthly temperature range −0.122, 0.274; isothermality 0.192, 0; temperature seasonality −0.245, −0.13; maximum temperature warmest month 0.269, 0; minimum temperature coldest month 0.336, 0; temperature annual range −0.214, 0.123; mean temperature wettest quarter 0.274, 0; mean temperature driest quarter 0.336, 0; mean temperature warmest quarter 0.282, 0; mean temperature coldest quarter 0.339, 0; annual precipitation 0.187, −0.292; precipitation wettest month 0.226, 0; precipitation driest month 0, −0.477; precipitation seasonality 0, 0.421; precipitation wettest quarter 0.224, 0; precipitation driest quarter 0, −0.482; precipitation warmest quarter −0.113, −0.184; precipitation coldest quarter 0.132, −0.331. Main result of this analysis: the first two principal components (PCs) accounted for 64% of the climatic variance, with highest loadings for temperature variables along PC 1 (minimum temperature coldest month, mean temperature driest quarter, mean temperature coldest quarter) and precipitation variables along PC 2 (precipitation driest month, precipitation seasonality, precipitation of driest quarter). The PCA suggests that within the D. minutus group, the climatic niches (i.e., 19 bioclimatic temperature and precipitation dimensions) are rather similar, even when the two main groups are compared (i.e. lineages 1–18 vs. 19–43). Some lineages from the periphery of the known geographic distribution of the group, including lineages 1–18, are weakly separated (lineages 9, 11–13 15, 16). (DOCX) [file pone.0103958.s003.docx]

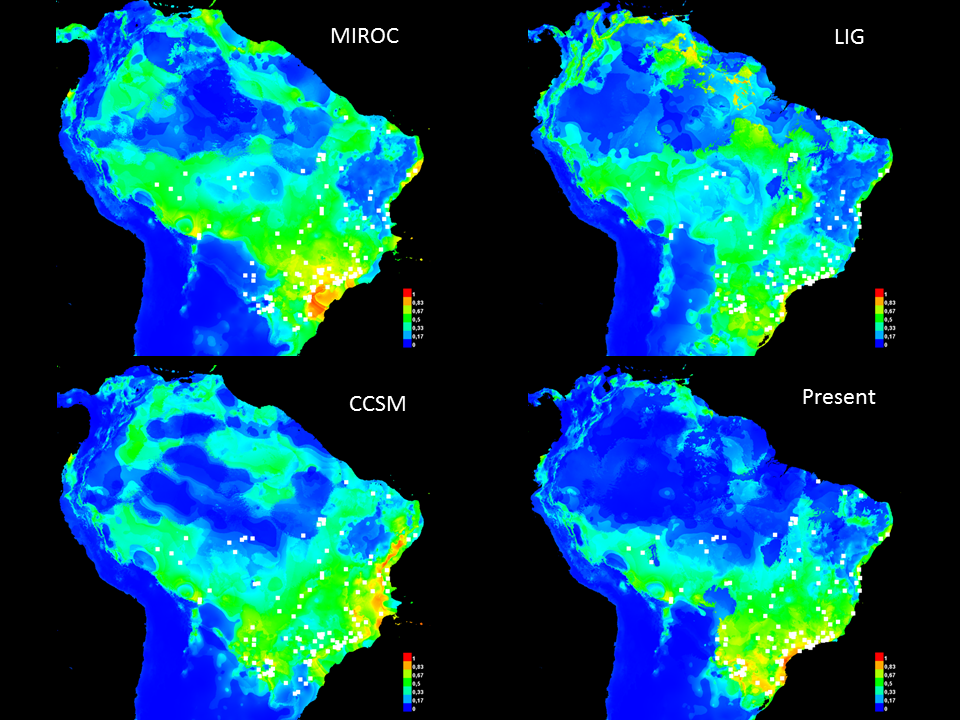

Supplement: Figure S4 — Spatial distribution models used as resistance layers in the Circuit Scape analysis. (DOCX) [file pone.0103958.s004.docx]

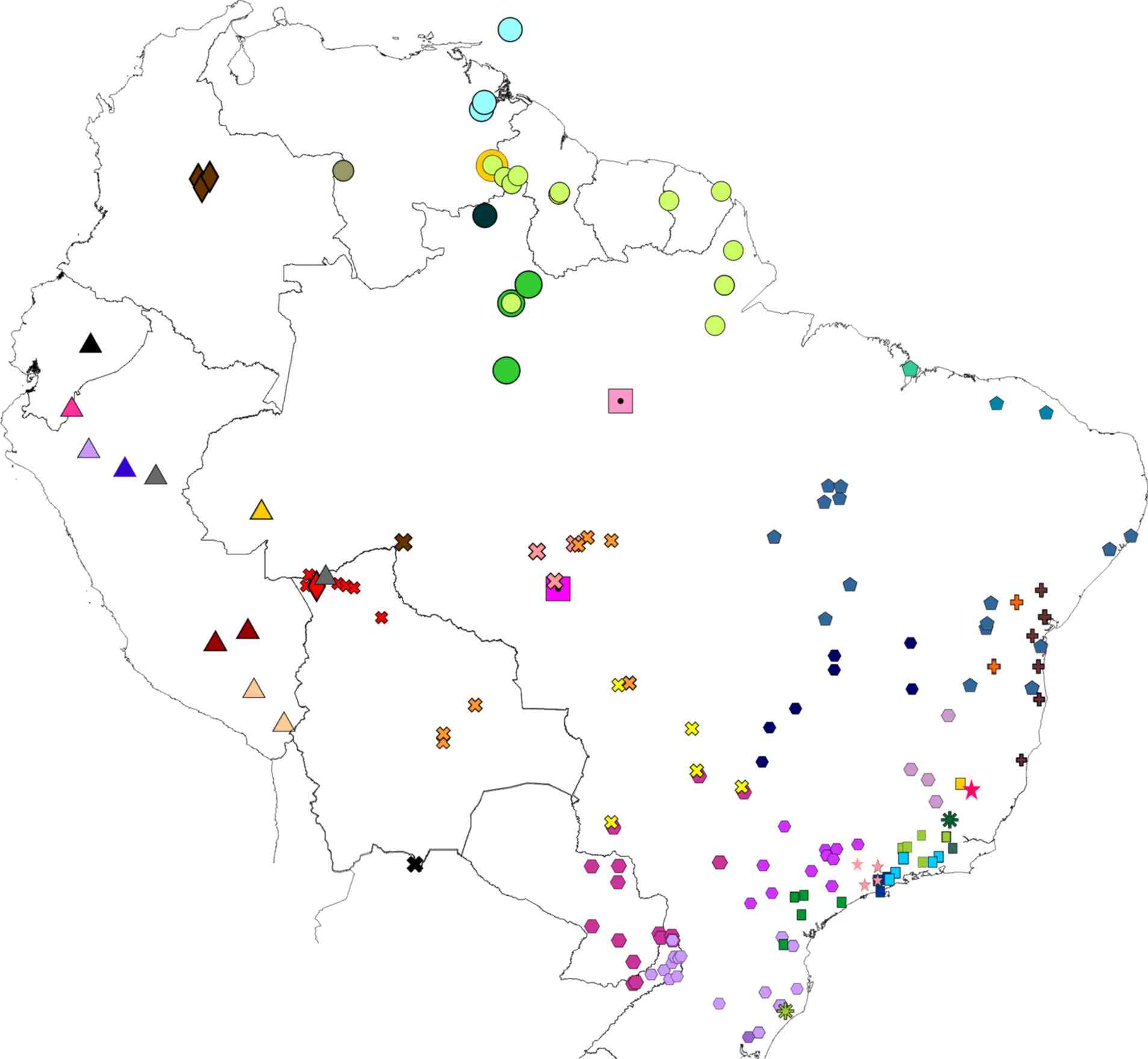

Supplement: Figure S5 — Distribution of all mitochondrial lineages. Map showing the distribution of all mitochondrial lineages in the Dendropsophus minutus group as revealed by this study. Symbols refer to those used in Figs. 1–3. (DOCX) [file pone.0103958.s005.docx]
