# Supplementary material for: High Levels of Diversity Uncovered in a Widespread Nominal Taxon: Continental Phylogeography of the Neotropical Tree Frog Dendropsophus minutus
Source: PLoS One. 2014 Sep 10;9(9):e103958. doi: 10.1371/journal.pone.0103958 (PMC4160190; doi:10.1371/journal.pone.0103958)
Supplement: Table S2 — Species used in the substitution rate estimation with respective Genbank accession numbers. (DOCX) [file pone.0103958.s007.docx]

|  | GenBank accession number | Species |  | GenBank accession number | Species |  | GenBank accession number | Species |  |
| --- | --- | --- | --- | --- | --- | --- | --- | --- | --- |
|  | AY843559 | *Acris crepitans* |  | AY843725 | *Hylomantis lemur* |  | AY843709 | *Osteocephalus taurinus* |  |
|  | AY843560 | *Acris gryllus* |  | AY843618 | *Hyloscirtus charazani* |  | AY843710 | *Osteopilus crucialis* |  |
|  | AY843563 | *Agalychnis callidryas* |  | AY843620 | *Hyloscirtus colymba* |  | AY843711 | *Osteopilus dominicensis* |  |
|  | AY843566 | *Anotheca spinosa* |  | AY843650 | *Hyloscirtus palmeri* |  | AY843712 | *Osteopilus septentrionalis* |  |
|  | AY843567 | *Aparasphenodon brunoi* |  | AY843599 | *Hypsiboas andinus* |  | AY843713 | *Osteopilus vastus* |  |
|  | AY843596 | *Aplastodiscus albosignatus* |  | AY843606 | *Hypsiboas benitezi* |  | AY843714 | *Pachymedusa dacnicolor* |  |
|  | AY843604 | *Aplastodiscus arildae* |  | AY843610 | *Hypsiboas boans* |  | AY843715 | *Phasmahyla cochranae* |  |
|  | AY843614 | *Aplastodiscus callipygius* |  | AY843613 | *Hypsiboas calcaratus* |  | AY843716 | *Phasmahyla guttata* |  |
|  | AY843617 | *Aplastodiscus cavicola* |  | AY843621 | *Hypsiboas crepitans* |  | AY843721 | *Phyllodytes luteolus* |  |
|  | AY843568 | *Aplastodiscus cochranae* |  | AY843628 | *Hypsiboas geographicus* |  | AY843722 | *Phyllodytes sp.* |  |
|  | AY843669 | *Aplastodiscus eugenioi* |  | AY843631 | *Hypsiboas guentheri* |  | AY843723 | *Phyllomedusa bicolor* |  |
|  | AY843638 | *Aplastodiscus leucopygius* |  | AY843632 | *Hypsiboas heilprini* |  | AY843724 | *Phyllomedusa hypochondrialis* |  |
|  | AY843569 | *Aplastodiscus perviridis* |  | AY843636 | *Hypsiboas lanciformis* |  | AY843726 | *Phyllomedusa tarsius* |  |
|  | AY843685 | *Aplastodiscus weygoldti* |  | AY843637 | *Hypsiboas lemai* |  | AY843727 | *Phyllomedusa tetraploidea* |  |
|  | AY843570 | *Argenteohyla siemersi* |  | AY843639 | *Hypsiboas lundii* |  | AY843728 | *Phyllomedusa tomopterna* |  |
|  | AY843677 | *Bokermannohyla aff. alvarengai* |  | AY843644 | *Hypsiboas microderma* |  | AY843675 | *Plectrohyla aff. thorectes* |  |
|  | AY843676 | *Bokermannohyla aff. pseudopseudis* |  | AY843648 | *Hypsiboas multifasciatus* |  | AY843602 | *Plectrohyla arborescandens* |  |
|  | AY843641 | *Bokermannohyla martinsi* |  | AY843670 | *Hypsiboas nympha* |  | AY843609 | *Plectrohyla bistincta* |  |
|  | AY843673 | *Bokermannohyla sp.* |  | AY843651 | *Hypsiboas pardalis* |  | AY843615 | *Plectrohyla calthula* |  |
|  | AY843674 | *Bokermannohyla sp.* |  | AY843655 | *Hypsiboas polytaenius* |  | AY843622 | *Plectrohyla cyclada* |  |
|  | AY843612 | *Bromeliohyla bromeliacia* |  | AY843657 | *Hypsiboas raniceps* |  | AY843730 | *Plectrohyla glandulosa* |  |
|  | AY843649 | *Charadrahyla nephila* |  | AY843660 | *Hypsiboas roraima* |  | AY843731 | *Plectrohyla guatemalensis* |  |
|  | AY843679 | *Charadrahyla taeniopus* |  | AY843662 | *Hypsiboas rufitelus* |  | AY843732 | *Plectrohyla matudai* |  |
|  | AY843578 | *Corythomantis greeningi* |  | AY843779 | *Hypsiboas semilineatus* |  | AY843734 | *Pseudacris cadaverina* |  |
|  | AY843562 | *Cruziohyla calcarifer* |  | AY843667 | *Hypsiboas sibleszi* |  | AY843735 | *Pseudacris crucifer* |  |
|  | DQ116870 | *Cyclorana alboguttata* |  | AY843671 | *Hypsiboas sp.* |  | AY843736 | *Pseudacris ocularis* |  |
|  | AY843580 | *Cyclorana australis* |  | AY843656 | *Isthmohyla pseudopuma* |  | AY843737 | *Pseudacris regilla* |  |
|  | AY843597 | *Dendropsophus anceps* |  | AY843659 | *Isthmohyla rivularis* |  | AY843738 | *Pseudacris triseriata* |  |
|  | AY843607 | *Dendropsophus berthalutzae* |  | DQ830818 | *Isthmohyla tica* |  | EF153005 | *Pseudis bolbodactyla* |  |
|  | AY843608 | *Dendropsophus bipunctatus* |  | DQ830809 | *Isthmohyla zeteki* |  | EF152997 | *Pseudis cardosoi* |  |
|  | AY843611 | *Dendropsophus brevifrons* |  | AY843706 | *Itapotihyla langsdorffii* |  | EF153003 | *Pseudis fusca* |  |
|  | AY843616 | *Dendropsophus carnifex* |  | AY843691 | *Litoria aurea* |  | AY843739 | *Pseudis minuta* |  |
|  | AY843624 | *Dendropsophus ebraccatus* |  | DQ116859 | *Litoria bicolor* |  | AY843740 | *Pseudis paradoxa* |  |
|  | AY843629 | *Dendropsophus giesleri* |  | AY843692 | *Litoria caerulea* |  | EF153008 | *Pseudis paradoxa* |  |
|  | AY843635 | *Dendropsophus labialis* |  | DQ116874 | *Litoria chloris* |  | EF153004 | *Pseudis tocantins* |  |
|  | AY843640 | *Dendropsophus marmoratus* |  | DQ116865 | *Litoria coplandi* |  | AY843623 | *Ptychohyla dendrophasma* |  |
|  | AY843643 | *Dendropsophus microcephalus* |  | DQ116868 | *Litoria dahlii* |  | AY843744 | *Ptychohyla euthysanota* |  |
|  | AY843647 | *Dendropsophus miyatai* |  | DQ116860 | *Litoria fallax* |  | AY843745 | *Ptychohyla hypomykter* |  |
|  | AY843652 | *Dendropsophus parviceps* |  | AY843693 | *Litoria freycineti* |  | AY843746 | *Ptychohyla leonhardschultzei* |  |
|  | AY843658 | *Dendropsophus rhodopeplus* |  | DQ283222 | *Litoria genimaculata* |  | AY843747 | *Ptychohyla sp.* |  |
|  | AY843661 | *Dendropsophus rubicundulus* |  | DQ116872 | *Litoria gilleni* |  | AY843748 | *Ptychohyla spinipollex* |  |
|  | AY843663 | *Dendropsophus sanborni* |  | DQ116876 | *Litoria gracilenta* |  | AY843749 | *Ptychohyla zophodes* |  |
|  | AY843664 | *Dendropsophus sarayacuensis* |  | DQ116863 | *Litoria inermis* |  | AY843752 | *Scarthyla goinorum* |  |
|  | AY843666 | *Dendropsophus seniculus* |  | AY843694 | *Litoria infrafrenata* |  | AY843753 | *Scinax acuminatus* |  |
|  | AY843680 | *Dendropsophus triangulum* |  | DQ283204 | *Litoria lesueurii* |  | AY843754 | *Scinax berthae* |  |
|  | AY843683 | *Dendropsophus walfordi* |  | AY843695 | *Litoria meiriana* |  | AY843755 | *Scinax boulengeri* |  |
|  | AY843583 | *Duellmanohyla rufioculis* |  | DQ116855 | *Litoria microbelos* |  | AY843756 | *Scinax catharinae* |  |
|  | AY843584 | *Duellmanohyla soralia* |  | DQ283218 | *Litoria nannotis* |  | AY843757 | *Scinax elaeochroa* |  |
|  | AY843777 | *Ecnomiohyla miliaria* |  | DQ116862 | *Litoria nasuta* |  | AY843758 | *Scinax fuscovarius* |  |
|  | AY843645 | *Ecnomiohyla miotympanum* |  | DQ116864 | *Litoria pallida* |  | AY843759 | *Scinax nasicus* |  |
|  | AY843619 | *Exerodonta chimalapa* |  | DQ116857 | *Litoria peronii* |  | AY843760 | *Scinax squalirostris* |  |
|  | AY843642 | *Exerodonta melanomma* |  | DQ116858 | *Litoria rothii* |  | AY843761 | *Scinax staufferi* |  |
|  | AY843653 | *Exerodonta perkinsi* |  | DQ116856 | *Litoria rubella* |  | AY843681 | *Scinax uruguayus* |  |
|  | AY843686 | *Exerodonta xera* |  | DQ116873 | *Litoria splendida* |  | AY843762 | *Smilisca baudinii* |  |
|  | AY843598 | *Hyla andersonii* |  | DQ116861 | *Litoria tornieri* |  | AY843763 | *Smilisca cyanosticta* |  |
|  | AY843600 | *Hyla annectans* |  | DQ116866 | *Litoria watjulumensis* |  | AY843743 | *Smilisca fodiens* |  |
|  | AY843601 | *Hyla arborea* |  | DQ116875 | *Litoria xanthomera* |  | AY843764 | *Smilisca phaeota* |  |
|  | AY843603 | *Hyla arenicolor* |  | EF152999 | *Lysapsus caraya* |  | AY843765 | *Smilisca puma* |  |
|  | AY843605 | *Hyla avivoca* |  | AY843696 | *Lysapsus laevis* |  | DQ830822 | *Smilisca sila* |  |
|  | AF315161 | *Hyla chinensis* |  | AY843697 | *Lysapsus limellum* |  | AY843766 | *Sphaenorhynchus dorisae* |  |
|  | AY330892 | *Hyla cinerea* |  | EF153002 | *Lysapsus limellum* |  | AY843770 | *Tepuihyla edelcae* |  |
|  | AY843625 | *Hyla euphorbiacea* |  | AY843646 | *Megastomatohyla mixe* |  | DQ830811 | *Tlalocohyla godmani* |  |
|  | AY843626 | *Hyla eximia* |  | AY843672 | *Myersiohyla inparquesi* |  | AY843654 | *Tlalocohyla picta* |  |
|  | AY843627 | *Hyla femoralis* |  | AY843634 | *Myersiohyla kanaima* |  | AY843668 | *Tlalocohyla smithii* |  |
|  | AY843630 | *Hyla gratiosa* |  | AY843781 | *Nyctimantis rugiceps* |  | AY843717 | *Trachycephalus hadroceps* |  |
|  | AY843633 | *Hyla japonica* |  | DQ283220 | *Nyctimystes dayi* |  | AY843771 | *Trachycephalus jordani* |  |
|  | GQ916810 | *Hyla meridionalis* |  | AY843702 | *Nyctimystes kubori* |  | AY843718 | *Trachycephalus mesophaeus* |  |
|  | AY843665 | *Hyla savignyi* |  | AY843703 | *Nyctimystes narinosus* |  | AY843772 | *Trachycephalus nigromaculatus* |  |
|  | AY843678 | *Hyla squirella* |  | AY843701 | *Nyctimystes pulcher* |  | AY843719 | *Trachycephalus resinifictrix* |  |
|  | AY843682 | *Hyla versicolor* |  | AY843705 | *Osteocephalus cabrerai* |  | AY843774 | *Triprion petasatus* |  |
|  | AY843684 | *Hyla walkeri* |  | AY843707 | *Osteocephalus leprieurii* |  | DQ830815 | *Triprion spatulata* |  |
|  | AY843687 | *Hylomantis granulosa* |  | AY843708 | *Osteocephalus oophagus* |  | AY843775 | *Xenohyla truncata* |  |
|  |  |  |  |  |  |  |  |  |  |
