# Supplementary material for: High Levels of Diversity Uncovered in a Widespread Nominal Taxon: Continental Phylogeography of the Neotropical Tree Frog Dendropsophus minutus
Source: PLoS One. 2014 Sep 10;9(9):e103958. doi: 10.1371/journal.pone.0103958 (PMC4160190; doi:10.1371/journal.pone.0103958)
Supplement: Table S4 — List of primers used in this study with respective anealing temperatures. (DOCX) [file pone.0103958.s009.docx]

| Gene | Primer Name | Sequence (5' - 3') | Anealing temperature | Source |
| --- | --- | --- | --- | --- |
| 16S | 16S-MG-F | CTYGTACCTTTYGCATCATGRTTTA | 50°C | This study |
| 16S | 16s-Isch1-R | CCTGATCCAACATCGAGGTCGT |  | Gehara et al. 2013 |
|  |  |  |  |  |
| COI | dgLCO1490 | GGTCAACAAATCATAAAGAYATYGG | 48°C | ([Folmer et al., 1994](#_ENREF_10); [Meyer et al., 2005](#_ENREF_16)) |
| COI | dgHCO2198 | TAAACTTCAGGGTGACCAAARAAYCA |  |  |
