# Supplementary material for: High Levels of Diversity Uncovered in a Widespread Nominal Taxon: Continental Phylogeography of the Neotropical Tree Frog Dendropsophus minutus
Source: PLoS One. 2014 Sep 10;9(9):e103958. doi: 10.1371/journal.pone.0103958 (PMC4160190; doi:10.1371/journal.pone.0103958)
Supplement: Table S5 — Localities and coordinates used in the Spatial Distribution Modeling. (DOCX) [file pone.0103958.s010.docx]

| **Locality** | **State/Province** | **Country** | **longitude** | **latitude** |
| --- | --- | --- | --- | --- |
| Orán | Salta | Argentina | -64.375194 | -22.725361 |
| Cobija | Pando | Bolivia | -68.750000 | -11.033300 |
| Barracon | Pando | Bolivia | -66.933300 | -11.559100 |
| Los Lagos | Beni, Yucuma | Bolivia | -65.810933 | -12.771967 |
| Los Lagos | Beni, Yucuma | Bolivia | -65.809400 | -12.772867 |
| Uruçuí-Una | Piauí | Brazil | -44.974444 | -8.922500 |
| Jequitinhonha | Minas Gerais | Brazil | -41.000000 | -16.433300 |
| Linhares | Espírito Santo | Brazil | -39.890100 | -19.371900 |
| Linhares | Espírito Santo | Brazil | -39.890099 | -19.371851 |
| Camacan | Bahia | Brazil | -39.496100 | -15.419700 |
| Prado | Bahia | Brazil | -39.216700 | -17.350000 |
| Ituberá | Bahia | Brazil | -39.150000 | -13.733300 |
| Porto Seguro | Bahia | Brazil | -39.083300 | -16.433300 |
| Ilhéus | Bahia | Brazil | -39.033300 | -14.816700 |
| Jequitinhonha | Minas Gerais | Brazil | -41.003300 | -16.435500 |
| Boa Nova | Bahia | Brazil | -40.166700 | -14.366700 |
| Monte Verde | Minas Gerais | Brazil | -46.144700 | -22.755300 |
| Biritiba Mirin | São Paulo | Brazil | -45.866700 | -23.633300 |
| São Luis do Paraitinga | São Paulo | Brazil | -45.555300 | -23.026400 |
| Resende | Rio de Janeiro | Brazil | -44.450000 | -22.466700 |
| Lima Duarte | Minas Gerais | Brazil | -43.800000 | -21.850000 |
| Maromba | Rio de Janeiro | Brazil | -43.775300 | -22.852200 |
| Além Paraiba | Rio de Janeiro | Brazil | -42.703889 | -21.887778 |
| Parque Nacional do Caparaó | Minas Gerais | Brazil | -41.874700 | -20.433100 |
| Nova Friburgo | Rio de Janeiro | Brazil | -42.533300 | -22.266700 |
| Piraquara | Paraná | Brazil | -49.063300 | -25.441700 |
| Ribeirão Branco | São Paulo | Brazil | -48.765600 | -24.220800 |
| Iporanga | São Paulo | Brazil | -48.593100 | -24.585600 |
| Guapiara | São Paulo | Brazil | -48.532800 | -24.185000 |
| Itanhaém | São Paulo | Brazil | -46.788900 | -24.183100 |
| São Sebastião | São Paulo | Brazil | -45.409700 | -23.760000 |
| Bairro Alto | São Paulo | Brazil | -45.352500 | -23.473600 |
| Ubatuba | São Paulo | Brazil | -45.071100 | -23.433900 |
| São José do Barreiro | São Paulo | Brazil | -44.661667 | -23.041667 |
| Duque de Caxias | Rio de Janeiro | Brazil | -43.308300 | -22.786700 |
| Petrópolis. | Rio de Janeiro | Brazil | -43.178600 | -22.505000 |
| Aripuanã | Mato Grosso | Brazil | -59.459400 | -10.166700 |
| Estação Ecológica Iquê | Mato Grosso | Brazil | -58.740833 | -11.377778 |
| Porto Velho | Rondônia | Brazil | -65.049181 | -9.591318 |
| Camino de Santa Cruz a Porongo | Santa Cruz | Bolivia | -63.306300 | -17.855700 |
| Ibañez | Santa Cruz | Bolivia | -63.289983 | -17.517200 |
| San Sebastián | Santa Cruz | Bolivia | -62.000050 | -16.359633 |
| Nova Bandeirantes | Mato Grosso | Brazil | -57.861944 | -9.813889 |
| Apiacás | Mato Grosso | Brazil | -57.449200 | -9.543600 |
| Paranaíta | Mato Grosso | Brazil | -56.476700 | -9.664700 |
| Chapada dos Guimarães | Mato Grosso | Brazil | -55.750000 | -15.420000 |
| Bonito | Mato Grosso do Sul | Brazil | -56.481900 | -21.121100 |
| Rondonópolis | Mato Grosso | Brazil | -56.190000 | -15.540600 |
| Alto Araguaia | Mato Grosso | Brazil | -53.214900 | -17.309700 |
| Paraíso | Mato Grosso do Sul | Brazil | -53.012200 | -19.017500 |
| Paranaíba | Mato Grosso do Sul | Brazil | -51.190800 | -19.677200 |
| Alcântara | Maranhão | Brazil | -44.400000 | -2.400000 |
| Caseara | Tocantins | Brazil | -49.955600 | -9.278300 |
| Palmeirante | Tocantins | Brazil | -47.925800 | -7.860000 |
| Paranã | Tocantins | Brazil | -47.883700 | -12.616200 |
| Araguaína/Babaçulândia | Tocantins | Brazil | -47.756900 | -7.204700 |
| Goiatins | Tocantins | Brazil | -47.314200 | -7.710000 |
| Carolina | Maranhão | Brazil | -47.261500 | -7.229400 |
| Estação Ecológica Serra Geral do Tocantins | Tocantins | Brazil | -46.885600 | -11.220800 |
| São João do Paraíso | Minas Gerais | Brazil | -42.014400 | -15.314900 |
| Andaraí | Bahia | Brazil | -41.325900 | -12.802200 |
| Chapada Diamantina | Bahia | Brazil | -41.166700 | -11.972200 |
| Ubajara | Ceará | Brazil | -40.933300 | -3.850000 |
| Pacoti | Ceará | Brazil | -38.923300 | -4.225000 |
| Campo Alegre | Alagoas | Brazil | -36.350800 | -9.781900 |
| Passo de Camarajibe | Alagoas | Brazil | -35.493300 | -9.238300 |
| Vicosa | Minas Gerais | Brazil | -42.881944 | -20.753889 |
| Cainguas | Missiones | Argentina | -54.952778 | -27.086667 |
| Cainguas | Missiones | Argentina | -54.952222 | -27.087714 |
| Guaraní | Missiones | Argentina | -54.426250 | -26.917861 |
| Guaraní | Missiones | Argentina | -54.200000 | -27.283330 |
| San Pedro | Missiones | Argentina | -54.103694 | -26.631306 |
| Iguazú | Missiones | Argentina | -54.086528 | -25.701111 |
| Guaraní | Missiones | Argentina | -53.900000 | -27.166670 |
| San Pedro | Missiones | Argentina | -53.839167 | -26.425833 |
| Mato Castelhano | Rio Grande do Sul | Brazil | -52.191700 | -28.278300 |
| Sapiranga | Rio Grande do Sul | Brazil | -51.006900 | -29.638100 |
| São Francisco de Paula | Rio Grande do Sul | Brazil | -50.583600 | -29.448100 |
| Lages | Santa Catarina | Brazil | -50.326100 | -27.816100 |
| Balsa Nova | Paraná | Brazil | -49.640000 | -25.580000 |
| Serra do Rio do Rastro | Rio Grande do Sul | Brazil | -49.624700 | -28.336900 |
| São Francisco de Paula | Rio Grande do Sul | Brazil | -49.145472 | -25.896257 |
| Rancho Queimado | Santa Catarina | Brazil | -49.033300 | -27.683300 |
| Ortigueira | Paraná | Brazil | -50.916700 | -24.200000 |
| Assis | São Paulo | Brazil | -50.412200 | -22.661700 |
| Pinhalão | Paraná | Brazil | -50.050000 | -23.783300 |
| Nova Itapirema | São Paulo | Brazil | -49.543100 | -21.076100 |
| Itirapina | São Paulo | Brazil | -48.445000 | -22.885800 |
| São Carlos | São Paulo | Brazil | -47.890833 | -22.017778 |
| Brotas | São Paulo | Brazil | -47.822800 | -22.252800 |
| Araçoiaba da Serra | São Paulo | Brazil | -47.614200 | -23.505300 |
| Rio Claro | São Paulo | Brazil | -47.561400 | -22.411400 |
| Pirassununga | São Paulo | Brazil | -47.425800 | -21.996100 |
| Poços de Caldas | Minas Gerais | Brazil | -46.566700 | -21.800000 |
| Quirinópolis | Goiás | Brazil | -50.451700 | -18.448300 |
| Jandaia | Goiás | Brazil | -50.146100 | -17.048600 |
| Campo Limpo de Goiás | Goiás | Brazil | -49.100000 | -16.283300 |
| São João da Aliança | Goiás | Brazil | -47.521900 | -14.707940 |
| Alto Paraíso de Goiás | Goiás | Brazil | -47.510000 | -14.132500 |
| Jaborandi | Bahia | Brazil | -44.432800 | -13.619400 |
| Januária | Minas Gerais | Brazil | -44.362600 | -15.495600 |
| Cerrados del Tagatiyá | Concepción | Paraguay | -57.363000 | -22.686000 |
| Emboscada | Cordillera | Paraguay | -57.355000 | -25.123000 |
| Capitán Bado | Amambay | Paraguay | -56.306000 | -23.335000 |
| Colonia Independencia | Guairá | Paraguay | -56.259000 | -25.695000 |
| Estancia Pirá Potrero | Amambay | Paraguay | -56.258000 | -22.672000 |
| Alto Verá | Itapúa | Paraguay | -55.675000 | -26.575000 |
| Candelaria | Missiones | Argentina | -55.576389 | -27.395000 |
| Itabó | Alto Paraná | Paraguay | -54.631000 | -25.414000 |
| Iguazú | Missiones | Argentina | -54.550000 | -25.600000 |
| Iguazú | Missiones | Argentina | -54.116670 | -25.508890 |
| Iguazú | Missiones | Argentina | -54.086390 | -25.701110 |
| Teodoro Sampaio | São Paulo | Brazil | -52.167500 | -22.532500 |
| Candelaria | Missiones | Argentina | -50.681944 | -22.462778 |
| Curvelo | Minas Gerais | Brazil | -44.416700 | -18.750000 |
| Santana do Riacho | Minas Gerais | Brazil | -43.714400 | -19.168900 |
| Catas Altas | Minas Gerais | Brazil | -43.400000 | -20.066700 |
| Grãomogol | Minas Gerais | Brazil | -42.900000 | -16.566700 |
