# Supplementary material for: High Levels of Diversity Uncovered in a Widespread Nominal Taxon: Continental Phylogeography of the Neotropical Tree Frog Dendropsophus minutus
Source: PLoS One. 2014 Sep 10;9(9):e103958. doi: 10.1371/journal.pone.0103958 (PMC4160190; doi:10.1371/journal.pone.0103958)
Supplement: Methods S1 — Supplementary methods. (DOC) [file pone.0103958.s011.doc]

**Methods S1**

**Laboratory methods:** Molecular genetic work was carried out in various laboratories, as follows. DNA extraction was carried out following a standard salt extraction protocol using DNeasy Blood & Tissue Kit (Qiagen,Valencia, CA, USA), DNA extraction kit (Promega), or with a standard phenol-choloform protocol implemented with an AutoGenprep 965 (AutoGen) automated DNA isolation robot. We used polymerase chain reaction (PCR) to amplify two fragments of mitochondrial DNA (mtDNA): one fragment of the 16S ribosomal RNA gene and the cytochrome oxidase subunit I (COI) barcode fragment, using primers and annealing temperatures described in **Table S4**. PCR products were cleaned using enzymatic purifications . Sequencing of purified PCR products was performed with standard Sanger sequencing technique either by using ABI Big Dye version 3.1 (ABI, Foster City, USA and Darmstadt, Germany) and resolved in an automated sequencer at Genomic Engenharia Corp. (São Paulo, Brazil) and Zoological Institute of TU-Braunschweig (Braunschweig, Germany); or by sending to an external service platform (Macrogen, South Korea). Chromatograms were checked using the following software: Sequencer 4.5, MacClade 4.08, CodonCode Aligner v.3.x (CodonCode Corporation, Dedham, MA, USA).

**Phylogeography:** The recently developed phylogeographical method by Lemey et al. (2010) integrates geographical information and genealogical inference to estimate geographical locality of internal nodes. Dispersal patterns are then inferred by reconstructing ancestral latitude and longitude assuming a Brownian diffusion model. The software is a Bayesian implementation of a model similar to the likelihood dispersal model implemented in the program Phylomapper . The employment of the model in a Bayesian framework is beneficial because topology uncertainty can be incorporated on the ancestral reconstruction and posterior density of node localities can be calculated . Another improvement by Lemey et al. (2010) is the implementation of the Relaxed Random Walk model (RRW) which allows the dispersal rate to vary across branches, a model analogous to the Relaxed Clock model. Using this approach we performed a RRW phylogeographical analysis to estimate the geographical origin of the *D. minutus* group, possible dispersal routes, and approximately when it dispersed to different regions of the South American continent. We favored the RRW due to the wide geographical range of the samples analyzed. As some mtDNA lineages have wider ranges than others we assumed that a model that allow variation of dispersal rate would better represent our data. The method is implemented in the software BEAST 1.7.2 which estimates tree topology, node and root height and geographical locality of nodes simultaneously. In this and all analyses using BEAST we also used the Beagle library version 1.0 to improve computational efficiency. We used as locality traits GPS coordinates collected in the field, when available. When not available, we used as traits the coordinates of the municipality were the particular sample was collected, which was retrieved from online Gazetteers. For this analysis we used the same dataset described in the section above without the outgroup. We partitioned the sequences to allow distinct models and parameter values for each gene while linking tree topology.

**Spatial modelling:** As our point locality data might represent multiple cryptic species or lineages, the Species Distribution Model (SDM) obtained could be affected by the magnitude of the differences in environmental niche between the different lineages. To assess the extent of climatic niche diversification in the *Dendropsophus minutus* group, we plotted the first two PC scores of a Principal Component Analysis (PCA) calculated from the values of the 19 bioclimatic variables representing current climatic conditions, with spatial resolution of 2.5 seconds . Values of the bioclimatic variables at the 170 localities with coordinate records were obtained using the ‘Extract Values by Points’ function using DIVA-GIS 7.3.0 , and, after deletion of duplicates, processed in a PCA using R version 2.15.3 (R Foundation for Statistical Computing; http://www.r-project.org/). Clustering of localities of lineages in multivariate climatic space could be indicative of niche similarity . The PCA results indicated a high level of niche similarity, except for the deepest lineages (lineages 1–18, see Results). In light of these results, we took a conservative approach and constructed a niche model only for a monophyletic clade composed of closely related lineages, which represent the *D. minutus* complex (lineages 19–43, see Results).

Before performing SDM, we created a correlation matrix among all bioclimatic variables. Then, we selected from the dataset six spatially uncorrelated (Pearson’s *r* < 0.7) and more biologically meaningful variables (annual mean temperature, temperature seasonality, temperature annual range, mean temperature of warmest quarter, annual precipitation, precipitation of the wettest month). To geographically balance the sampling we created a fishnet of 0.5 degrees over the extent of the geographical coordinates and kept only one point per grid, this resulted in a subset of 118 distinct localities (**Table S5**), diminishing the potential bias of heavily sampled areas. We used Maxent version 3.3.3a , to construct the SDM using the bioclimatic layers and the point locality information. Maxent algorithm was run for 500 iterations with a convergence threshold of 0.00001, 10000 background points and expressing the resulting suitability values in logistic format. The Maxtent modeling approach uses presence-only data to estimate the maximum entropy distribution that satisfies constraints imposed by the data and ranks among the best modeling techniques based on presence-only data . The efficiency of the model was evaluated with the AUC statistic calculated from 100 models iteratively calculated after separating a random 25% of the data for testing. The final model showed good predictive capacity (AUC value: 0.89). Model results were then projected to three different past climatic scenarios to define areas of suitable climate. One projection for the last interglacial period (LIG), 120,000 years ago; and two projections for the Last Glacial Maximum (LGM), 21,000 year ago [CCSM3 (Community Climate System Model); MIROC (Model of Interdisciplinary Research on Climate)]. Past climate scenarios were downloaded from www.diva-gis.org .

.

**Time constraints used estimating substitution rate:** We constrained three nodes with uniform priors based on fossil evidence as follows: 1) most recent common ancestor (MRCA) of *Acris* and *Pseudacris* to 15 Ma or older; 2) MRCA of *Hyla squirella* and *H. cinerea* to 15 Ma or older; and 3) MRCA of all *Hyla* as 33 Ma or older. Given the uncertainty inherent in calibration number 3 , we performed separate analyses with and without this latter calibration. The biogeographic evidence of the time of separation between South America and Australia was used to calibrate the divergence between Phyllomedusinae and Pelodryadinae. The complete separation of east Antarctica and Australia happen at approximately 35.5 Ma, while the last landbridge between South America and west Antarctica broke up at around 32–28 Ma with the opening of the Drake passage and the formation of the South Circumpolar Current . We took into consideration two potential pitfalls of using this biogeographical evidence for a forth calibration: 1) the divergence between the two groups could have predated the separation of the continents; 2) overseas dispersals may happen after land separation . Therefore, we used a normal prior with a mean of 32 Ma and standard deviation of 6 Ma to allow a higher probability for sampling around this value (quantiles: 5% = 22.13 Ma; 95% = 41.87 Ma) while avoiding hard boundaries. Before constraining the heights of the nodes we performed a preliminary run without constrain to verify if the resulting topology would recover the nodes correspondent to the calibration scheme. As this was confirmed we proceeded with the approach. The analysis was carried out in the program BEAST 1.7.2 and it was repeated three times to check for convergence. We used a GTR++I mutation model inferred with jModelTest 0.1 and Akaike information criterium – AIC , a Yule Process speciation prior and an uncorrelated lognormal relaxed clock . We used the posterior density of the parameter *ucld.mean* as an estimation of the substitution rate of the mitochondrial 16S gene in hylids.

**References**

Akaike, H., 1974. A new look at the statistical model identification. IEEE Transactions on Automatic Control 19, 716–723.

Broennimann, O., Fitzpatrick, M.C., Pearman, P.B., Petitpierre, B., Pellissier, L., Yoccoz, N.G., Thuiller, W., Fortin, M.-J., Randin, C., Zimmermann, N.E., Graham, C.H., Guisan, A., 2011. Measuring ecological niche overlap from occurrence and spatial environmental data. Global Ecol Biogeogr 21, 481–497.

Bruford, M.W., Hanotte, O., Brookfield, J.F.Y., Burke, T., 1992. Single-locus and multilocus DNA fingerprint. Molecular Genetic Analysis of Populations: A Practical Approach. IRL Press, Oxford, pp. 225–270.

Busby, J.R., 1991. BIOCLIM - a bioclimatic analysis and prediction system. Nature Conservation: cost effective biological surveys and data analysis, pp. 64-68.

Drummond, A.J., Ho, S.Y.W., Phillips, M.J., Rambaut, A., 2006. Relaxed phylogenetics and dating with confidence. PLoS Biol 4, e88.

Drummond, A.J., Suchard, M.A., Xie, D., Rambaut, A., 2012. Bayesian phylogenetics with BEAUti and the BEAST 1.7. Mol Biol Evol.

Elith, J., Graham, C.H., Anderson, R.P., Dudík, M., Ferrier, S., Guisan, A., Hijmans, R.J., Huettmann, F., Leathwick, J.R., Lehmann, A., Li, J., Lohmann, L.G., Loiselle, B.A., Manion, G., Moritz, C., Nakamura, M., Nakazawa, Y., Overton, J.M., Peterson, A.T., Phillips, S.J., Richardson, K., Scachetti-Pereira, R., Schapire, R.E., Soberón, J., Williams, S., Wisz, M.S., Zimmermann, N.E., 2006. Novel methods improve prediction of species’ distributions from occurrence data. Ecography 29, 129-151.

Elith, J., Leathwick, J.R., 2009. Species distribution models: Ecological explanation and prediction across space and time. Annu Rev Ecol Evol Syst 40, 677–697.

Faivovich, J., Haddad, C.F.B., Garcia, P.C.A., Frost, D.R., Campbell, J.A., Wheeler, W.C., 2005. Systematic review of the frog family Hylidae, with special reference to Hylinae: phylogenetic analysis and taxonomic revision. Bull Am Mus Nat Hist 294, 240 pp.

Folmer, O., Black, M., Hoeh, W., Lutz, R., Vrijenhoek, R., 1994. DNA primers for amplification of mitochondrial cytochrome c oxidase subunit I from diverse metazoan invertebrates. Mol Mar Biol Biotechnol 3, 294-299.

Hijmans, R.J., Cameron, S.E., Parra, J.L., Jones, P.G., Jarvis, A., 2005. Very high resolution interpolated climate surfaces for global land areas. Int J Climatol 25, 1965-1978.

Hijmans, R.J., Cruz, J.M., Rojas, R., Guarino, L., 2001. DIVA-GIS, version 1.4. A geographic information system for the management and analysis of genetic resources data. Manual. International Potato Center and International Plant Genetic Resources Institute, Lima, Peru.

Lemey, P., Rambaut, A., Welch, J.J., Suchard, M.A., 2010. Phylogeography takes a relaxed random walk in continuous space and time. Mol Biol Evol 27, 1877-1885.

Lemmon, A.R., Lemmon, E.M., 2008. A likelihood framework for estimating phylogeographic history on a continuous landscape. Syst Biol 57, 544-561.

McLoughlin, S., 2001. The breakup history of Gondwana and its impact on pre-Cenozoic floristic provincialism. Aust J Bot 49, 271–300.

Meyer, C.P., Geller, J.B., Paulay, G., 2005. Fine scale endemism on coral reefs: archipelagic differentiation in turbinid gastropods. Evolution 59, 113-125.

Phillips, S.J., Anderson, R.P., Schapire, R.E., 2006. Maximum entropy modeling of species geographic distributions. Ecol Model 190, 231–259.

Phillips, S.J., Dudík, M., 2008. Modeling of species distributions with Maxent: new extensions and a comprehensive evaluation. Ecography 31, 161-175.

Posada, D., 2008. jModelTest: phylogenetic model averaging. Mol Biol Evol 25, 1253-1256.

Vences, M., Vieites, D.R., Glaw, F., Brinkmann, H., Kosuch, J., Veith, M., Meyer, A., 2003. Multiple overseas dispersal in amphibians. Proc Biol Sci 270, 2435-2442.

Werle, E., Schneider, C., Renner, M., Volker, M., Fiehn, W., 1994. Convenient single-step, one tube purification of PCR products for direct sequencing. Nucleic Acids Res 22, 4354-4355.
